# National trends of unmet healthcare needs and risk factors by household income level, 2010 to 2022: A Nationwide cross-sectional study in South Korea

**DOI:** 10.1097/MD.0000000000047143

**Published:** 2026-01-23

**Authors:** Hyunjee Kim, Jaeyu Park, Jinyoung Jeong, Saiah Kim, Hayeon Lee, Hyeon Jin Kim, Yejun Son, Soeun Kim, Sooji Lee, Kyeongmin Lee, Hyesu Jo, Yesol Yim, Masoud Rahmati, Damiano Pizzol, Lee Smith, Ho Geol Woo, Dong Keon Yon

**Affiliations:** a Center for Digital Health, Medical Science Research Institute, Kyung Hee University College of Medicine, Seoul, South Korea; b Department of Precision Medicine, Kyung Hee University College of Medicine, Seoul, South Korea; c Department of Medicine, Kyung Hee University College of Medicine, Seoul, South Korea; d Department of Molecular Bioscience, University of South Florida, Tampa, FL; e Department of Biomedical Engineering, Kyung Hee University, Yongin, South Korea; f Department of Regulatory Science, Kyung Hee University, Seoul, South Korea; g Health Service Research and Quality of Life Center (CEReSS), Assistance Publique-Hôpitaux de Marseille, Aix-Marseille Université, Marseille, France; h Department of Physical Education and Sport Sciences, Faculty of Literature and Human Sciences, Lorestan University, Khoramabad, Iran; i Department of Physical Education and Sport Sciences, Faculty of Literature and Humanities, Vali-E-Asr University of Rafsanjan, Rafsanjan, Iran; j Health Unit Eni, Maputo, Mozambique; k Health Unit, Eni, San Donato Milanese, Italy; l Centre for Health, Performance and Wellbeing, Anglia Ruskin University, Cambridge, UK; m Department of Public Health, Faculty of Medicine, Biruni University, Istanbul, Turkey; n Department of Neurology, Kyung Hee University Medical Center, Kyung Hee University College of Medicine, Seoul, South Korea; o Department of Pediatrics, Kyung Hee University College of Medicine, Seoul, South Korea.

**Keywords:** epidemiology, risk factors, South Korea, unmet healthcare needs

## Abstract

Socio-economic status plays a critical role in shaping unmet healthcare needs, and the COVID-19 pandemic has further intensified these disparities; however, research to date remains insufficient. Therefore, this study aims to analyze unmet healthcare needs by household income using large-scale longitudinal data (2010–2022) including pre- and post- pandemic differences. This large-scale study (n = 2,628,584) utilized nationwide data from the Korea Community Health Survey (KCHS) conducted between 2010 and 2022, administered by the Korea Disease Control and Prevention Agency. The analysis employed complex, weighted sampling to examine trends in unmet healthcare needs, with a specific focus on changes during the COVID-19 pandemic. Weighted logistic regression models were used to calculate odds ratios and β differences (β_diff_) between the pre-pandemic (2010–2019) and pandemic (2020–2022) periods. In total, 2,628,584 individuals participated in the KCHS from 2010 to 2022, comprising 1,454,129 males (55.3%) and 1,174,455 females (44.7%). Before the pandemic, there was a consistent decline in the prevalence of unmet healthcare needs. However, following the onset of the pandemic, unmet healthcare needs increased (β_diff,_ low-level of household income: 1.66 [95% CI, 1.41–1.92]; mid-level of household income: 0.88 [95% CI, 0.77–0.99]; high-level of household income: 0.71 [95% CI, 0.57–0.85]). Overall, households with lower incomes exhibited higher levels of unmet healthcare needs compared to those with higher incomes (low household income: 14.8 [95% CI, 13.91–14.24]; high household income: 8.45 [95% CI, 8.34–8.55]). Additionally, the disparity in healthcare access due to income differences was more pronounced among older individuals, those with lower educational attainment, and those with lower subjective health status. Our analysis found that older adults in low-income households consistently faced higher rates of unmet healthcare needs. The reversal of a pre-pandemic trend toward reducing healthcare gaps highlights the urgent need for targeted interventions to address socio-economic disparities.

## 1. Introduction

Access to health services is primarily measured through unmet healthcare needs, which influence individual health outcomes and overall quality of life, so improving them is essential for reducing socio-economic disparities.^[1]^ However, despite efforts from governments, healthcare disparities continue, especially among low-income households with high unmet healthcare needs.^[2]^ Previous literature has also suggested that those with low economic status have approximately 4 times higher odds of experiencing unmet healthcare needs.^[3,4]^ Therefore, it is imperative to assess unmet healthcare needs through the lens of household income.

These disparities became more pronounced following the COVID-19 pandemic. The pandemic had a negative impact on employment and working hours, resulting in increased unemployment and income reductions due to involuntary reductions in work hours.^[5]^ In particular, low-skilled, low-wage workers – among whom remote work was largely infeasible – faced disproportionately high risks of job loss. This, in turn, exacerbated income inequality, leading to a substantial increase in unmet healthcare needs among low-income households.^[6]^ However, previous studies only focused on specific age or sex groups, without considering the unique challenges posed by the pandemic or involving sufficiently large participant groups.^[7,8]^ In addition, many of these studies mainly analyzed the initial emergence of COVID-19.^[3,9]^ In the contemporary healthcare field, rapid technological advancements are transforming medical systems and workflows.^[10–12]^ However, despite these innovations, fundamental disparities in healthcare access persist, particularly among low-income populations. Therefore, addressing unmet healthcare needs remains a crucial task for achieving equity and sustainability within health systems.

Therefore, this study aims to analyze trends in unmet medical needs by household income using large-scale longitudinal data from 2010 to 2022 and to present various determinants and differences before and after the pandemic. Based on these results, our study suggests strengthening public health campaigns for preventive care, expanding financial assistance, and enhancing insurance coverage to alleviate cost barriers and reduce out-of-pocket expenditures.

## 2. Methods

### 2.1. Study population

This large-scale study utilized nationwide data from the Korea Community Health Survey (KCHS) conducted between 2010 and 2022.^[13,14]^ The Korea Disease Control and Prevention Agency (KDCA) conducted this survey to produce community health statistics among adults aged 19 years and older.^[13,14]^ The survey encompassed a broad range of health-related behaviors, including unmet healthcare needs and household income levels.^[13,14]^ Trained interviewers visited selected households to collect data on health behaviors, body measurements, age, sex, and region.^[15]^ To ensure accurate statistical representation, the sampling procedure employed population stratification based on 253 community health centers and systematically selected households according to the number classified by the type of residence.^[16]^ The KCHS data for the years covered by this study were anonymized, and written informed consent was obtained from all participants before participation. The study protocol was approved by the Institutional Review Board of the KDCA (2010-02CON-22-P, 2011-05CON04-C, 2012-07CON-01-2C, 2013-06EXP-01-3C, 2014-08EXP-09-4CA, and 2016-10-01-TA). Written informed consent was obtained from all participants before they participated in the study. The research was conducted in accordance with ethical guidelines established by the relevant national and institutional review boards for human subject research and adhered to the principles of the Declaration of Helsinki, as revised in 2008.^[17]^

### 2.2. Ascertainment of unmet healthcare needs

The study divided the survey period into 6 segments: 2010 to 2011, 2012 to 2013, 2014 to 2015, 2016 to 2017, 2018 to 2019 (pre-pandemic), and 2020 to 2022 (during the pandemic). Participants were asked if they had required medical care (testing or treatment) in the past year but did not receive it, with response options of “yes” or “no.”

### 2.3. Covariates

This study included covariates to account for additional factors that could influence the actual association. These covariates included age (categorized as 19–30, 31–40, 41–50, 51–60, 61–70, 71–80, and over 81), sex (male and female), region of residence (urban and rural),^[18]^ body mass index (BMI; underweight, normal, overweight, and obese), educational attainment (high school or lower education and college or higher education), smoking status (smoker, ex-smoker, and nonsmoker), frequency of alcohol consumption (nondrinker and 1 day/month, 2 to 9 days/month, over 10 days/month), and subjective health-level (high, middle, low). BMI was subdivided based on self-reported height and weight into 4 categories (underweight [<18.5 kg/m^2^], normal [18.5–22.9 kg/m^2^], overweight [23.0–24.9 kg/m^2^], and obese [≥25.0 kg/m^2^]) according to the Asia-Pacific guidelines.^[19]^

BMI was reclassified into 2 categories: “under and normal” and “over and obese.” To assess participants’ perceptions of their health status, they were asked, “How would you rate your overall health?” with response options ranging from “very good” to “very poor.” Their answers were grouped into 3 categories: “high” (including “very good” and “good”), “middle,” and “low” (including “poor” and “very poor”).

### 2.4. Statistical analyses

To examine the long-term national trend in the prevalence of unmet healthcare needs, we conducted a weighted, complex-sampling analysis. Our study assesses trends in unmet healthcare needs over the past 12 years, with a focus on the COVID-19 pandemic period. We analyzed shifts in trends using the difference in β (β_diff_) from 2010 to 2019 to 2020 to 2022 (before and during the pandemic). Additionally, we derived weighted odds ratios and 95% confidence intervals from weighted logistic regression models.^[20]^ Based on these analyses, we compared high-level income and low-level income using middle-level income as a reference to illustrate the difference in unmet healthcare needs between the lowest and highest income groups compared to the middle-income group. Finally, we obtained the ratio of odds ratios to estimate the interaction term for each risk factor. This ratio was calculated using OR values obtained before and during the pandemic,^[21]^ allowing us to interpret which groups became more vulnerable to meeting healthcare needs during the pandemic. All statistical analyses were conducted using SAS software (version 9.4; SAS Inc., Cary). The test was 2-sided, and *P*-values <.05 were considered statistically significant.

## 3. Results

In total, 2,628,584 individuals were included in the KCHS between 2010 and 2022. Among them, 1,454,129 (55.3%) were male and 1,174,455 (44.7%) were female. Table 1 suggests that a significant number of participants had a mid-level of household income (n = 1,582,115; 19.1%). This study sample had an average age of 53.33 years (SD = 17.46). Participants with low household income had a notably higher average age of 68.96 years (SD = 13.88), whereas those with high household income had a younger average age of 45.29 years (SD = 14.22) (Table S1, Supplemental Digital Content, https://links.lww.com/MD/R123).

**Table 1:** Baseline characteristics of Koreans from 2010 to 2022, based on KCHS data (N = 2,628,584), crude numbers and percent.

	Total	Low-level of household income	Mid-level of household income	High-level of household income
Overall, n	2,628,584	503,262	1582,115	543,207
Crude rate (95% CI)				
Age, mean (SD)	53.33 (17.46)	68.96 (13.88)	51.12 (16.46)	45.29 (14.22)
Age, years, n (%)				
19–30	310,758 (15.06)	15,535 (3.09)	198,094 (12.52)	97,129 (17.88)
31–40	373,808 (17.26)	11,114 (2.21)	262,814 (16.61)	99,880 (18.39)
41–50	466,497 (19.95)	21,907 (4.35)	296,826 (18.76)	147,764 (27.20)
51–60	507,986 (19.16)	49,948 (9.92)	328,032 (20.73)	130,006 (23.93)
61–70	458,033 (14.94)	125,368 (24.91)	288,358 (18.23)	44,307 (8.16)
71–80	374,103 (10.23)	196,471 (39.04)	160,533 (10.15)	17,099 (3.15)
Over 81	137,399 (3.40)	82,919 (16.48)	47,458 (3.00)	7022 (1.29)
Sex, n (%)				
Male	1,174,455 (44.95)	181,821 (36.13)	738,703 (46.69)	253,931 (46.75)
Female	1,454,129 (55.05)	321,441 (63.87)	843,412 (53.31)	289,276 (53.25)
Region of residence, n (%)				
Urban	1,497,817 (81.88)	165,410 (32.87)	928,532 (58.69)	403,875 (74.35)
Rural	1,130,767 (18.12)	337,852 (67.13)	653,583 (41.31)	139,332 (25.65)
BMI group, n (%)*				
Underweight	220,025 (6.61)	92,597 (18.40)	98,019 (6.20)	29,409 (5.41)
Normal weight	1,089,653 (43.02)	194,262 (38.60)	658,393 (41.61)	236,998 (43.63)
Overweight	614,812 (23.49)	103,059 (20.48)	383,420 (24.23)	128,333 (23.63)
Obese	704,094 (26.87)	113,344 (22.52)	442,283 (27.96)	148,467 (27.33)
Education, n (%)				
Elementary school or lower education	659,266 (15.87)	332,335 (66.04)	297,103 (18.78)	29,828 (5.49)
Middle school	302,520 (9.88)	67,497 (13.41)	206,872 (13.08)	28,151 (5.18)
High school	757,855 (30.33)	68,016 (13.52)	535,871 (33.87)	153,968 (28.34)
College or higher education	908,943 (43.92)	35,414 (7.04)	542,269 (34.27)	331,260 (60.98)
Smoking status, n (%)				
Smoker	488,103 (18.94)	69,215 (13.75)	320,701 (20.27)	98,187 (18.08)
Ex-smoker	466,692 (17.22)	95,254 (18.93)	280,495 (17.73)	90,943 (16.74)
Nonsmoker	1,673,789 (63.84)	338,793 (67.32)	980,919 (62.00)	354,077 (65.18)
Alcohol consumption, n (%)				
Nondrinker and 1d/mo	1,567,215 (56.57)	385,979 (76.70)	907,736 (57.37)	273,500 (50.35)
2–9 d/mo	870,865 (37.02)	79,135 (15.72)	555,452 (35.11)	236,278 (43.50)
Over 10 d/mo	190,504 (6.42)	38,148 (7.58)	118,927 (7.52)	33,429 (6.15)
Health-level, n (%)				
High	998,280 (40.88)	95,374 (18.95)	632,359 (39.97)	270,547 (49.81)
Middle	1,083,304 (42.80)	167,568 (33.30)	689,229 (43.56)	226,507 (41.70)
Low	547,000 (16.32)	240,320 (47.75)	260,527 (16.47)	46,153 (8.50)

BMI = body mass index, CI = confidence interval, KCHS = Korea Community Health Survey.

* According to the Asian–Pacific guidelines, the BMI is divided into 4 groups: underweight (<18.5 kg/m^2^), normal (18.5–22.9 kg/m^2^), overweight (23.0–24.9 kg/m^2^), and obese (≥25.0 kg/m^2^).

**Figure 1.**
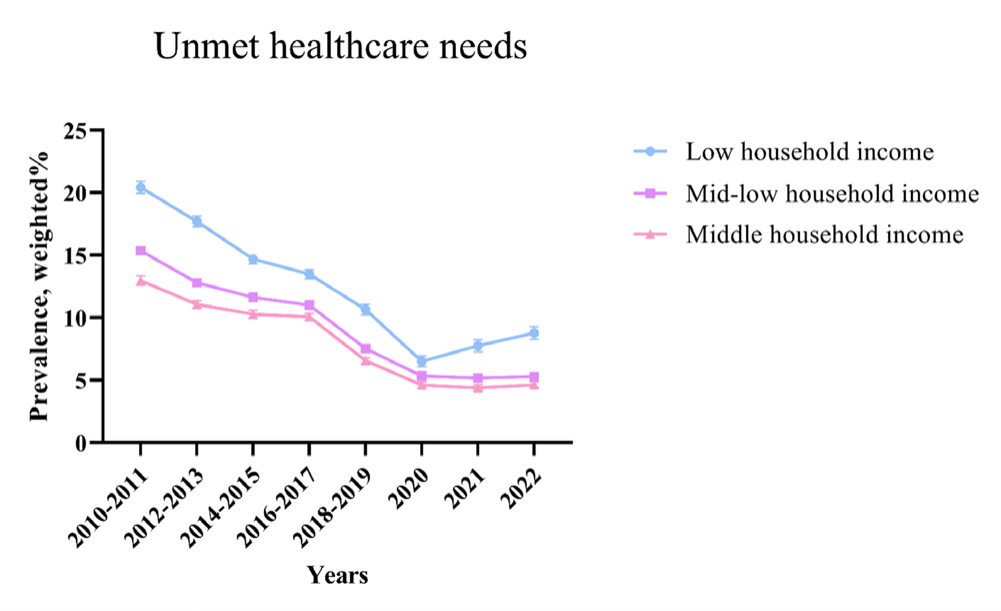
Nationwide trend in unmet healthcare needs prevalence over 13 yr (2010–2022) among 2,628,584 Korean adults, stratified by household income groups, 2010 to 2022.

Based on Figure 1 and Table 2, before the pandemic, there was a consistent decrease in the prevalence of unmet healthcare needs, with a significant reduction among low-income households (β, −2.37 [95% CI, −2.51 to −2.23]). Following the onset of COVID-19, unmet healthcare needs increased notably among low-income households (β_diff_, 1.66 [95% CI, 1.41–1.92]). The prevalence of unmet healthcare needs was highest among low-income households, followed by mid-level income households, and high-income households (low-level of household income: 14.08 [95% CI, 13.91–14.24]; mid-level of household income: 10.68 [95% CI, 10.60–10.76]; high-level of household income: 8.45 [95% CI, 8.34–8.55]) (Table S2, Supplemental Digital Content, https://links.lww.com/MD/R123).

**Table 2:** National trends in the prevalence of unmet healthcare needs and odds ratios β-coefficients before and during the COVID-19 pandemic, presented as weighted percentages with 95% confidence intervals, using data obtained from KCHS.

Variables			Total	Pre-pandemic					Pandemic			Trend in the pre-pandemic era, β (95% CI)	Trend in the pandemic era, β (95% CI)	Trend difference, β_diff_ (95% CI)
				2010–2011	2012–2013	2014–2015	2016–2017	2018–2019	2020	2021	2022			
Overall		Low-level of household income	14.08 (13.91–14.24)	20.43 (19.95–20.91)	17.70 (17.26–18.13)	14.69 (14.32–15.06)	13.48 (13.12–13.84)	10.65 (10.23–11.07)	6.51 (6.09–6.94)	7.75 (7.28–8.23)	8.79 (8.29–9.29)	**−2.37 (−2.51 to −2.23**)	**−0.71 (−0.92 to −0.49**)	**1.66 (1.41–1.92**)
		Mid-level of household income	10.68 (10.60–10.76)	15.37 (15.15–15.60)	12.80 (12.61–12.99)	11.64 (11.46–11.81)	11.00 (10.83–11.17)	7.54 (7.36–7.72)	5.34 (5.15–5.54)	5.17 (4.98–5.36)	5.31 (5.12–5.50)	**−1.70 (−1.77 to −1.64**)	**−0.82 (−0.91 to −0.73**)	**0.88 (0.77–0.99**)
		High-level of household income	8.45 (8.34–8.55)	12.97 (12.59–13.34)	11.08 (10.79–11.37)	10.29 (9.98–10.59)	10.07 (9.79–10.34)	6.58 (6.36–6.79)	4.62 (4.36–4.87)	4.39 (4.15–4.63)	4.64 (4.41–4.88)	**−1.41 (−1.50 to −1.32**)	**−0.70 (−0.81 to −0.60**)	**0.71 (0.57–0.85**)
Age, years	19–30	Low-level of household income	13.24 (12.59–13.89)	18.16 (16.37–19.94)	13.31 (11.66–14.96)	14.44 (13.03–15.85)	13.31 (11.88–14.74)	9.45 (7.74–11.17)	7.45 (5.21–9.70)	8.90 (6.41–11.39)	7.18 (4.78–9.58)	**−1.69 (−2.24 to −1.14**)	**−**0.62 (**−**1.58 to 0.34)	1.07 (**−**0.03 to 2.18)
		Mid-level of household income	12.04 (11.86–12.22)	16.07 (15.60–16.54)	12.99 (12.55–13.42)	12.96 (12.54–13.37)	12.35 (11.92–12.77)	8.84 (8.36–9.32)	6.20 (5.65–6.75)	5.69 (5.14–6.25)	6.35 (5.74–6.96)	**−1.44 (−1.60 to −1.29**)	**−1.00 (−1.25 to −0.74**)	**0.45 (0.15–0.74**)
		High-level of household income	9.04 (8.81–9.26)	13.62 (12.82–14.42)	11.46 (10.83–12.08)	11.82 (11.14–12.50)	11.02 (10.43–11.61)	6.78 (6.31–7.24)	4.54 (4.01–5.08)	3.93 (3.42–4.43)	4.72 (4.18–5.27)	**−1.47 (−1.66 to −1.27**)	**−0.81 (−1.04 to −0.58**)	**0.66 (0.36–0.96**)
	31–40	Low-level of household income	17.60 (16.64–18.56)	20.99 (19.02–22.96)	19.09 (16.76–21.41)	17.76 (15.74–19.77)	17.13 (14.75–19.51)	11.22 (8.24–14.19)	10.47 (6.18–14.77)	9.92 (6.67–13.18)	16.64 (11.22–22.06)	**−1.82 (−2.59 to −1.05**)	1.17 (**−**0.76 to 3.11)	**2.99 (0.91–5.07**)
		Mid-level of household income	13.66 (13.49–13.84)	18.00 (17.57–18.43)	15.01 (14.61–15.42)	14.45 (14.07–14.84)	14.20 (13.79–14.61)	9.66 (9.20–10.12)	7.05 (6.48–7.62)	6.19 (5.63–6.75)	6.16 (5.61–6.72)	**−1.63 (−1.78 to −1.49**)	**−1.31 (−1.55 to −1.06**)	**0.33 (0.05–0.61**)
		High-level of household income	10.84 (10.60–11.08)	15.89 (15.12–16.66)	13.39 (12.77–14.00)	13.47 (12.77–14.16)	13.19 (12.53–13.85)	8.31 (7.78–8.84)	5.93 (5.25–6.60)	4.82 (4.24–5.39)	5.21 (4.66–5.76)	**−1.57 (−1.77 to −1.37**)	**−1.16 (−1.41 to −0.90**)	**0.41 (0.09–0.74**)
	41–50	Low-level of household income	19.24 (18.51–19.97)	24.60 (22.93–26.28)	21.84 (20.04–23.64)	19.47 (17.88–21.06)	18.33 (16.62–20.03)	14.15 (11.76–16.55)	9.58 (6.95–12.21)	10.71 (8.14–13.29)	15.90 (12.52–19.28)	**−2.35 (−2.96 to −1.74**)	0.12 (**−**1.20 to 1.44)	**2.47 (1.01–3.92**)
		Mid-level of household income	11.95 (11.78–12.11)	16.44 (16.01–16.87)	13.65 (13.26–14.04)	12.15 (11.81–12.50)	12.06 (11.69–12.43)	8.53 (8.11–8.94)	6.70 (6.16–7.23)	5.83 (5.33–6.33)	5.94 (5.42–6.47)	**−1.69 (−1.82 to −1.55**)	**−0.97 (−1.20 to −0.75**)	**0.71 (0.45–0.97**)
		High-level of household income	9.03 (8.84–9.21)	13.17 (12.55–13.79)	11.47 (10.96–11.97)	10.12 (9.60–10.64)	10.30 (9.80–10.79)	7.33 (6.92–7.74)	4.94 (4.45–5.42)	5.19 (4.68–5.69)	5.29 (4.80–5.77)	**−1.29 (−1.45 to −1.13**)	**−0.73 (−0.93 to −0.52**)	**0.57 (0.30–0.83**)
	51–60	Low-level of household income	18.40 (17.88–18.92)	24.26 (22.96–25.57)	21.39 (20.08–22.70)	18.69 (17.59–19.78)	17.23 (16.11–18.35)	15.54 (13.79–17.29)	7.89 (6.48–9.30)	11.92 (9.88–13.96)	13.60 (11.50–15.70)	**−2.23 (−2.67 to −1.78**)	**−**0.69 (**−**1.58 to 0.19)	**1.53 (0.54–2.52**)
		Mid-level of household income	9.98 (9.83–10.13)	13.53 (13.09–13.96)	12.32 (11.93–12.71)	10.43 (10.09–10.76)	9.92 (9.59–10.25)	7.71 (7.33–8.09)	5.51 (5.09–5.93)	5.94 (5.48–6.40)	6.42 (5.94–6.89)	**−1.38 (−1.51 to −1.26**)	**−0.52 (−0.72 to −0.32**)	**0.86 (0.63–1.10**)
		High-level of household income	6.74 (6.57–6.92)	10.17 (9.46–10.88)	9.09 (8.54–9.63)	7.75 (7.22–8.29)	7.99 (7.51–8.48)	5.42 (5.05–5.78)	4.23 (3.77–4.68)	4.39 (3.93–4.85)	4.74 (4.31–5.17)	**−1.09 (−1.26 to −0.93**)	**−0.26 (−0.44 to −0.07**)	**0.84 (0.59–1.08**)
	61–70	Low-level of household income	13.27 (12.98–13.56)	19.05 (18.30–19.80)	16.41 (15.67–17.15)	12.65 (12.01–13.29)	12.18 (11.50–12.85)	9.91 (9.07–10.75)	6.69 (5.84–7.54)	8.45 (7.42–9.47)	8.72 (7.64–9.81)	**−2.30 (−2.55 to −2.05**)	**−**0.37 (**−**0.81 to 0.07)	**1.93 (1.42–2.44**)
		Mid-level of household income	6.51 (6.38–6.64)	10.56 (10.08–11.04)	8.96 (8.56–9.36)	7.01 (6.67–7.35)	6.45 (6.13–6.77)	5.33 (5.04–5.62)	3.72 (3.40–4.04)	4.11 (3.78–4.45)	4.22 (3.90–4.53)	**−1.28 (−1.40 to −1.15**)	**−0.38 (−0.52 to −0.23**)	**0.90 (0.71–1.09**)
		High-level of household income	4.33 (4.08–4.58)	7.28 (6.20–8.35)	6.45 (5.55–7.35)	4.74 (3.91–5.57)	5.65 (4.87–6.43)	3.60 (3.11–4.08)	2.81 (2.21–3.40)	3.35 (2.71–4.00)	2.59 (2.13–3.04)	**−0.83 (−1.08 to −0.59**)	**−0.28 (−0.50 to −0.06**)	**0.55 (0.23–0.88**)
	71–80	Low-level of household income	12.12 (11.89–12.35)	18.99 (18.26–19.71)	16.83 (16.17–17.49)	12.51 (11.99–13.03)	11.49 (10.96–12.02)	9.35 (8.80–9.91)	5.01 (4.48–5.54)	5.58 (5.00–6.16)	6.40 (5.78–7.02)	**−2.46 (−2.67 to −2.26**)	**−1.12 (−1.40 to −0.85**)	**1.34 (0.99–1.68**)
		Mid-level of household income	5.57 (5.40–5.73)	9.73 (9.07–10.39)	9.12 (8.55–9.68)	6.90 (6.42–7.38)	5.73 (5.29–6.16)	4.00 (3.68–4.31)	2.81 (2.44–3.17)	3.18 (2.80–3.55)	3.53 (3.17–3.90)	**−1.51 (−1.66 to −1.36**)	**−0.17 (−0.33 to −0.01**)	**1.34 (1.12–1.56**)
		High-level of household income	3.99 (3.60–4.38)	7.82 (6.29–9.34)	6.66 (5.37–7.94)	5.75 (4.25–7.25)	3.69 (2.79–4.60)	2.95 (2.15–3.74)	1.84 (1.03–2.65)	1.67 (0.87–2.48)	1.30 (0.77–1.83)	**−1.27 (−1.63 to −0.91**)	**−0.55 (−0.88 to −0.22**)	**0.72 (0.23–1.21**)
	Over 81	Low-level of household income	13.40 (13.04–13.76)	21.72 (20.35–23.10)	19.26 (18.07–20.45)	16.71 (15.68–17.74)	14.63 (13.76–15.50)	11.09 (10.32–11.86)	6.66 (5.84–7.47)	7.24 (6.41–8.06)	8.69 (7.86–9.53)	**−2.61 (−2.94 to −2.27**)	**−0.89 (−1.27 to −0.51**)	**1.71 (1.21–2.22**)
		Mid-level of household income	6.78 (6.46–7.11)	12.64 (11.21–14.07)	10.04 (8.89–11.18)	8.66 (7.61–9.71)	7.80 (6.91–8.69)	5.81 (5.13–6.49)	3.15 (2.48–3.83)	4.86 (4.01–5.70)	3.71 (3.13–4.29)	**−1.57 (−1.89 to −1.24**)	**−0.56 (−0.86 to −0.26**)	**1.01 (0.56–1.45**)
		High-level of household income	4.63 (4.04–5.23)	6.45 (4.29–8.61)	6.73 (4.83–8.64)	4.91 (3.04–6.79)	4.90 (3.25–6.55)	4.58 (3.25–5.91)	3.43 (1.79–5.07)	2.63 (1.30–3.96)	3.41 (2.09–4.74)	**−0.55 (−1.10 to −0.01**)	**−**0.47 (**−**1.12 to 0.17)	0.08 (**−**0.76 to 0.93)
Sex	Male	Low-level of household income	11.70 (11.46–11.93)	16.84 (16.18–17.51)	14.10 (13.47–14.72)	11.73 (11.22–12.23)	11.15 (10.63–11.67)	9.26 (8.63–9.90)	5.28 (4.66–5.91)	7.26 (6.45–8.07)	8.65 (7.84–9.47)	**−1.83 (−2.03 to −1.63**)	**−**0.27 (**−**0.60 to 0.07)	**1.56 (1.17–1.96**)
		Mid-level of household income	9.41 (9.31–9.50)	13.72 (13.44–14.00)	11.16 (10.92–11.41)	10.07 (9.85–10.29)	9.69 (9.46–9.91)	6.57 (6.34–6.81)	4.67 (4.41–4.93)	4.37 (4.13–4.62)	4.51 (4.26–4.76)	**−1.54 (−1.62 to −1.46**)	**−0.77 (−0.88 to −0.65**)	**0.77 (0.63–0.91**)
		High-level of household income	7.41 (7.28–7.55)	11.76 (11.29–12.24)	9.56 (9.19–9.93)	8.95 (8.56–9.35)	8.78 (8.43–9.13)	5.78 (5.51–6.06)	3.83 (3.49–4.16)	3.70 (3.38–4.02)	4.12 (3.82–4.42)	**−1.28 (−1.40 to −1.17**)	**−0.62 (−0.75 to −0.48**)	**0.66 (0.48–0.84**)
	Female	Low-level of household income	15.69 (15.48–15.89)	22.91 (22.33–23.49)	20.15 (19.61–20.69)	16.78 (16.31–17.26)	15.08 (14.64–15.53)	11.52 (11.02–12.02)	7.29 (6.77–7.82)	8.07 (7.52–8.62)	8.88 (8.28–9.48)	**−2.76 (−2.93 to −2.59**)	**−0.98 (−1.24 to −0.73**)	**1.78 (1.47–2.09**)
		Mid-level of household income	11.96 (11.86–12.06)	17.08 (16.78–17.38)	14.48 (14.22–14.74)	13.26 (13.02–13.50)	12.35 (12.11–12.58)	8.47 (8.23–8.71)	5.98 (5.72–6.25)	5.91 (5.64–6.19)	6.05 (5.79–6.32)	**−1.89 (−1.98 to −1.80**)	**−0.88 (−1.00 to −0.76**)	**1.01 (0.86–1.15**)
		High-level of household income	9.50 (9.35–9.64)	14.21 (13.71–14.70)	12.64 (12.24–13.04)	11.65 (11.23–12.06)	11.39 (11.00–11.77)	7.38 (7.08–7.68)	5.40 (5.03–5.76)	5.06 (4.71–5.40)	5.15 (4.84–5.47)	**−1.55 (−1.67 to −1.42**)	**−0.80 (−0.94 to −0.65**)	**0.75 (0.56–0.94**)

BMI = body mass index, CI = confidence interval, KCHS = Korea Community Health Survey.

The numbers in bold indicate a significant difference (*P* < .05).

* According to the Asian–Pacific guidelines, the BMI is divided into 4 groups: underweight (<18.5 kg/m^2^), normal (18.5–22.9 kg/m^2^), overweight (23.0–24.9 kg/m^2^), and obese (≥25.0 kg/m^2^).

Table 3 presents national trends in unmet healthcare needs before and during the COVID-19 pandemic. In the case of low-level of household income, an increasing trend is observed in the unmet healthcare needs ratio (OR, 2020: 1.21 [95% CI, 1.10–1.33]; 2021: 1.15 [95% CI, 1.05–1.26]). These patterns persisted across age, sex, region, and education levels (Table S3, Supplemental Digital Content, https://links.lww.com/MD/R123).

**Table 3:** Weighted odds ratios of unmet healthcare needs for each subsequent 2-yr survey period compared with the preceding period, stratified by age group and sex (weighted % [95% CI]), based on data from the KCHS (2010–2022).

Variables			2012–2013 versus 2010–2011 (reference)	*P*-value	2014–2015 versus 2012–2013 (reference)	*P*-value	2016–2017 versus 2014–2015 (reference)	*P*-value	2018–2019 versus 2016–2017 (reference)	*P*-value	2020 versus 2018–2019 (reference)	*P*-value	2021 versus 2020 (reference)	*P*-value	2022 versus 2021 (reference)	*P*-value
Overall		Low-level of household income	**0.84 (0.80–0.87**)	**<.0001**	**0.80 (0.77–0.84**)	**<.0001**	**0.91 (0.87–0.95**)	**<.0001**	**0.77 (0.73–0.81**)	**<.0001**	**0.58 (0.54–0.64**)	**<.0001**	**1.21 (1.10–1.33**)	**<.0001**	**1.15 (1.05–1.26**)	**.004**
		Mid-level of household income	**0.81 (0.79–0.83**)	**<.0001**	**0.90 (0.88–0.92**)	**<.0001**	**0.94 (0.92–0.96**)	**<.0001**	**0.66 (0.64–0.68**)	**<.0001**	**0.69 (0.66–0.73**)	**<.0001**	0.97 (0.91–1.03)	.261	1.03 (0.97–1.09)	.348
		High-level of household income	**0.84 (0.80–0.87**)	**<.0001**	**0.92 (0.88–0.96**)	**<.0001**	**0.98 (0.93–1.02**)	**.294**	**0.63 (0.60–0.66**)	**<.0001**	**0.69 (0.64–0.74**)	**<.0001**	0.95 (0.87–1.03)	.225	1.06 (0.98–1.15)	.158
Age, years	19–30	Low-level of household income	**0.69 (0.58–0.83**)	**<.0001**	1.10 (0.92–1.32)	.309	0.91 (0.77–1.08)	.269	**0.68 (0.54–0.86**)	**.001**	0.77 (0.52–1.15)	.204	1.21 (0.77–1.92)	.408	0.79 (0.49–1.28)	.339
		Mid-level of household income	**0.78 (0.74–0.82**)	**<.0001**	1.00 (0.95–1.05)	.925	**0.95 (0.90–1.00**)	**.046**	**0.69 (0.64–0.74**)	**<.0001**	**0.68 (0.61–0.77**)	**<.0001**	0.91 (0.79–1.06)	.237	1.12 (0.96–1.31)	.142
		High-level of household income	**0.82 (0.75–0.90**)	**<.0001**	1.04 (0.95–1.13)	.440	0.92 (0.85–1.01)	.079	**0.59 (0.53–0.65**)	**<.0001**	**0.66 (0.57–0.76**)	**<.0001**	0.86 (0.71–1.04)	.113	1.21 (1.00–1.47)	.046
	31–40	Low-level of household income	0.89 (0.74–1.07)	.219	0.92 (0.75–1.12)	.395	0.96 (0.77–1.19)	.697	**0.61 (0.43–0.86**)	**.005**	0.93 (0.54–1.59)	.780	0.94 (0.51–1.74)	.848	1.81 (0.98–3.34)	.057
		Mid-level of household income	**0.81 (0.77–0.84**)	**<.0001**	0.96 (0.91–1.00)	.052	0.98 (0.94–1.03)	.392	**0.65 (0.61–0.69**)	**<.0001**	**0.71 (0.64–0.79**)	**<.0001**	**0.87 (0.76–1.00**)	**.047**	1.00 (0.86–1.15)	.949
		High-level of household income	**0.82 (0.76–0.89**)	**<.0001**	1.01 (0.93–1.09)	.868	0.98 (0.90–1.06)	.575	**0.60 (0.55–0.65**)	**<.0001**	**0.70 (0.60–0.80**)	**<.0001**	**0.80 (0.67–0.96**)	**.016**	1.09 (0.91–1.29)	.351
	41–50	Low-level of household income	**0.86 (0.75–0.98**)	**.029**	0.87 (0.75–1.00)	.050	0.93 (0.80–1.08)	.336	**0.74 (0.59–0.92**)	**.008**	**0.64 (0.45–0.92**)	**.017**	1.13 (0.76–1.69)	.540	**1.58 (1.08–2.30**)	**.018**
		Mid-level of household income	**0.80 (0.77–0.84**)	**<.0001**	**0.88 (0.84–0.92**)	**<.0001**	0.99 (0.94–1.04)	.708	**0.68 (0.64–0.73**)	**<.0001**	**0.77 (0.69–0.86**)	**<.0001**	**0.86 (0.76–0.99**)	**.029**	1.02 (0.89–1.18)	.770
		High-level of household income	**0.85 (0.79–0.92**)	**<.0001**	**0.87 (0.81–0.94**)	**<.0001**	1.02 (0.94–1.10)	.637	**0.69 (0.64–0.75**)	**<.0001**	**0.66 (0.58–0.74**)	**<.0001**	1.05 (0.91–1.22)	.496	1.02 (0.88–1.18)	.789
	51–60	Low-level of household income	**0.85 (0.76–0.94**)	**.002**	**0.85 (0.76–0.94**)	**.002**	0.91 (0.81–1.01)	.069	0.88 (0.76–1.03)	.117	**0.47 (0.37–0.59**)	**<.0001**	**1.58 (1.19–2.11**)	**.002**	1.16 (0.89–1.51)	.260
		Mid-level of household income	**0.90 (0.85–0.95**)	**<.0001**	**0.83 (0.79–0.87**)	**<.0001**	**0.95 (0.90–1.00**)	**.037**	**0.76 (0.71–0.81**)	**<.0001**	**0.70 (0.63–0.77**)	**<.0001**	**1.08 (0.96–1.23**)	**.202**	1.09 (0.96–1.23)	.194
		High-level of household income	**0.88 (0.80–0.98**)	**.017**	**0.84 (0.76–0.93**)	**.001**	1.03 (0.94–1.14)	.516	**0.66 (0.60–0.73**)	**<.0001**	**0.77 (0.67–0.88**)	**<.0001**	1.04 (0.89–1.22)	.623	1.08 (0.93–1.26)	.299
	61–70	Low-level of household income	**0.83 (0.78–0.90**)	**<.0001**	**0.74 (0.68–0.80**)	**<.0001**	0.96 (0.88–1.04)	.316	**0.79 (0.71–0.89**)	**<.0001**	**0.65 (0.55–0.77**)	**<.0001**	**1.29 (1.07–1.55**)	**.008**	1.04 (0.86–1.24)	.703
		Mid-level of household income	**0.83 (0.78–0.89**)	**<.0001**	**0.77 (0.71–0.82**)	**<.0001**	**0.91 (0.85–0.99**)	**.018**	**0.82 (0.76–0.88**)	**<.0001**	**0.69 (0.62–0.77**)	**<.0001**	1.11 (0.97–1.27)	.120	1.03 (0.91–1.16)	.672
		High-level of household income	0.88 (0.71–1.09)	.243	**0.72 (0.57–0.92**)	**.007**	1.20 (0.95–1.52)	.124	**0.62 (0.51–0.76**)	**<.0001**	0.77 (0.59–1.01)	.063	1.20 (0.88–1.64)	.245	0.77 (0.58–1.01)	.057
	71–80	Low-level of household income	**0.86 (0.81–0.92**)	**<.0001**	**0.71 (0.66–0.76**)	**<.0001**	**0.91 (0.85–0.98**)	**.008**	**0.80 (0.73–0.86**)	**<.0001**	**0.51 (0.45–0.58**)	**<.0001**	1.12 (0.95–1.32)	.166	1.16 (0.98–1.36)	.079
		Mid-level of household income	0.93 (0.84–1.03)	.164	**0.74 (0.67–0.82**)	**<.0001**	**0.82 (0.73–0.91**)	**<.0001**	**0.69 (0.61–0.77**)	**<.0001**	**0.69 (0.59–0.82**)	**<.0001**	1.14 (0.94–1.38)	.193	1.12 (0.94–1.33)	.222
		High-level of household income	0.84 (0.63–1.13)	.252	0.86 (0.61–1.21)	.375	**0.63 (0.43–0.91**)	**.015**	0.79 (0.54–1.15)	.223	0.62 (0.36–1.06)	.081	0.91 (0.47–1.76)	.774	0.77 (0.40–1.50)	.445
	Over 81	Low-level of household income	**0.86 (0.77–0.96**)	**.008**	**0.84 (0.76–0.94**)	**.002**	**0.85 (0.77–0.95**)	**.002**	**0.73 (0.66–0.81**)	**<.0001**	**0.57 (0.48–0.68**)	**<.0001**	1.09 (0.92–1.30)	.316	**1.22 (1.04–1.43**)	**.015**
		Mid-level of household income	**0.77 (0.64–0.92**)	**.005**	0.85 (0.71–1.02)	.083	0.89 (0.74–1.07)	.222	**0.73 (0.61–0.87**)	**<.0001**	**0.53 (0.41–0.69**)	**<.0001**	**1.57 (1.16–2.11**)	**.003**	**0.76 (0.58–0.98**)	**.034**
		High-level of household income	1.05 (0.66–1.67)	.850	0.72 (0.43–1.19)	.194	1.00 (0.59–1.70)	.995	0.93 (0.58–1.48)	.764	0.74 (0.41–1.33)	.314	0.76 (0.37–1.55)	.451	1.31 (0.65–2.64)	.453
Sex	Male	Low-level of household income	**0.81 (0.76–0.87**)	**<.0001**	**0.81 (0.75–0.87**)	**<.0001**	0.94 (0.88–1.02)	.118	**0.81 (0.74–0.89**)	**<.0001**	**0.55 (0.47–0.64**)	**<.0001**	**1.40 (1.18–1.66**)	**<.0001**	**1.21 (1.03–1.42**)	**.018**
		Mid-level of household income	**0.79 (0.76–0.82**)	**<.0001**	**0.89 (0.86–0.92**)	**<.0001**	**0.96 (0.92–0.99**)	**.019**	**0.66 (0.63–0.69**)	**<.0001**	**0.70 (0.65–0.75**)	**<.0001**	0.93 (0.85–1.02)	.136	1.03 (0.94–1.13)	.489
		High-level of household income	**0.79 (0.75–0.84**)	**<.0001**	**0.93 (0.87–0.99**)	**.029**	0.98 (0.92–1.05)	.527	**0.64 (0.60–0.68**)	**<.0001**	**0.65 (0.58–0.72**)	**<.0001**	0.97 (0.85–1.10)	.602	1.12 (0.99–1.26)	.072
	Female	Low-level of household income	**0.85 (0.81–0.89**)	**<.0001**	**0.80 (0.76–0.84**)	**<.0001**	**0.88 (0.84–0.93**)	**<.0001**	**0.73 (0.69–0.78**)	**<.0001**	**0.60 (0.55–0.67**)	**<.0001**	1.12 (1.00–1.25)	.052	1.11 (1.00–1.24)	.060
		Mid-level of household income	**0.82 (0.80–0.85**)	**<.0001**	**0.90 (0.88–0.93**)	**<.0001**	**0.92 (0.89–0.95**)	**<.0001**	**0.66 (0.63–0.68**)	**<.0001**	**0.69 (0.65–0.73**)	**<.0001**	0.99 (0.92–1.06)	.743	1.03 (0.95–1.11)	.504
		High-level of household income	**0.87 (0.83–0.92**)	**<.0001**	**0.91 (0.86–0.96**)	**.001**	0.98 (0.92–1.03)	.377	**0.62 (0.59–0.66**)	**<.0001**	**0.72 (0.66–0.78**)	**<.0001**	0.93 (0.84–1.04)	.197	1.02 (0.92–1.13)	.697

BMI = body mass index, CI = confidence interval, KCHS = Korea Community Health Survey, OR = odds ratios.

The numbers in bold indicate significant differences (*P* < .05).

* According to the Asian–Pacific guidelines, the BMI is divided into 4 groups: underweight (<18.5 kg/m^2^), normal (18.5–22.9 kg/m^2^), overweight (23.0–24.9 kg/m^2^), and obese (≥25.0 kg/m^2^).

Table 4 presents a comparison of unmet healthcare needs between low- and high-household income levels, using the mid-level of household income as a benchmark. Low-income households consistently had higher rates of unmet healthcare needs compared to high-income households, both before and during the COVID-19 pandemic, with a ratio of odds ratio (ROR, low-level of household income: 1.32 [95% CI, 1.30–1.34]; high-level of household income: 0.79 [95% CI, 0.78–0.80]; reference mid-level of household income). This trend persisted even as overall unmet healthcare needs increased following the pandemic outbreak. Furthermore, it was observed that the disparity in unmet healthcare needs due to income differences was more pronounced with increasing age (19–30: low-level of household income: 1.10 [95% CI, 1.05–1.16]; high-level of household income: 0.75 [95% CI, 0.73–0.77]; over 81: low-level of household income: 1.98 [95% CI, 1.87–2.09]; high-level of household income: 0.68 [95% CI, 0.60–0.78]).

**Table 4:** Comparative analysis of Korean’s unmet healthcare needs indicators across various periods by household income levels, KCHS, 2010 to 2022.

				Pre-pandemic				Pandemic			
		Variables	Total	2010–2011	2012–2013	2014–2015	2016–2017	2018–2019	2020	2021	2022
Overall		Low-level of household income	**1.32 (1.30–1.34**)	**1.33 (1.29–1.37**)	**1.38 (1.34–1.42**)	**1.26 (1.23–1.30**)	**1.23 (1.19–1.26**)	**1.41 (1.35–1.48**)	**1.22 (1.13–1.31**)	**1.50 (1.40–1.61**)	**1.66 (1.55–1.77**)
		Mid-level of household income	1.00 (ref)	1.00 (ref)	1.00 (ref)	1.00 (ref)	1.00 (ref)	1.00 (ref)	1.00 (ref)	1.00 (ref)	1.00 (ref)
		High-level of household income	**0.79 (0.78–0.80**)	**0.84 (0.82–0.87**)	**0.87 (0.84–0.89**)	**0.88 (0.86–0.91**)	**0.92 (0.89–0.94**)	**0.87 (0.84–0.91**)	**0.87 (0.81–0.92**)	**0.85 (0.80–0.91**)	**0.87 (0.82–0.93**)
Age, years	19–30	Low-level of household income	**1.10 (1.05–1.16**)	**1.13 (1.02–1.25**)	1.03 (0.90–1.17)	**1.11 (1.01–1.24**)	1.08 (0.96–1.21)	1.07 (0.88–1.29)	1.20 (0.87–1.66)	**1.56 (1.15–2.12**)	1.13 (0.79–1.62)
		Mid-level of household income	1.00 (ref)	1.00 (ref)	1.00 (ref)	1.00 (ref)	1.00 (ref)	1.00 (ref)	1.00 (ref)	1.00 (ref)	1.00 (ref)
		High-level of household income	**0.75 (0.73–0.77**)	**0.85 (0.79–0.91**)	**0.88 (0.83–0.94**)	**0.91 (0.85–0.97**)	**0.89 (0.84–0.95**)	**0.77 (0.70–0.84**)	**0.73 (0.63–0.85**)	**0.69 (0.59–0.81**)	**0.74 (0.64–0.86**)
	31–40	Low-level of household income	**1.29 (1.22–1.36**)	**1.17 (1.06–1.29**)	**1.27 (1.12–1.44**)	**1.23 (1.09–1.38**)	**1.21 (1.05–1.39**)	1.16 (0.88–1.53)	1.49 (0.95–2.31)	**1.60 (1.13–2.28**)	**2.70 (1.90–3.83**)
		Mid-level of household income	1.00 (ref)	1.00 (ref)	1.00 (ref)	1.00 (ref)	1.00 (ref)	1.00 (ref)	1.00 (ref)	1.00 (ref)	1.00 (ref)
		High-level of household income	**0.79 (0.77–0.82**)	**0.88 (0.84–0.93**)	**0.89 (0.85–0.94**)	**0.93 (0.88–0.99**)	**0.93 (0.88–0.98**)	**0.86 (0.79–0.93**)	**0.84 (0.73–0.97**)	**0.78 (0.67–0.91**)	**0.85 (0.74–0.97**)
	41–50	Low-level of household income	**1.61 (1.55–1.68**)	**1.50 (1.39–1.61**)	**1.60 (1.47–1.75**)	**1.60 (1.47–1.75**)	**1.52 (1.38–1.68**)	**1.66 (1.39–1.98**)	**1.43 (1.07–1.92**)	**1.84 (1.42–2.38**)	**2.68 (2.12–3.38**)
		Mid-level of household income	1.00 (ref)	1.00 (ref)	1.00 (ref)	1.00 (ref)	1.00 (ref)	1.00 (ref)	1.00 (ref)	1.00 (ref)	1.00 (ref)
		High-level of household income	**0.76 (0.74–0.78**)	**0.80 (0.76–0.85**)	**0.84 (0.80–0.89**)	**0.83 (0.79–0.88**)	**0.85 (0.81–0.90**)	**0.86 (0.80–0.93**)	**0.74 (0.65–0.84**)	0.89 (0.78–1.01)	0.89 (0.78–1.01)
	51–60	Low-level of household income	**1.84 (1.79–1.90**)	**1.79 (1.68–1.91**)	**1.74 (1.62–1.86**)	**1.79 (1.68–1.92**)	**1.74 (1.62–1.87**)	**2.02 (1.78–2.28**)	**1.43 (1.18–1.74**)	**2.01 (1.66–2.43**)	**2.12 (1.78–2.52**)
		Mid-level of household income	1.00 (ref)	1.00 (ref)	1.00 (ref)	1.00 (ref)	1.00 (ref)	1.00 (ref)	1.00 (ref)	1.00 (ref)	1.00 (ref)
		High-level of household income	**0.68 (0.66–0.70**)	**0.75 (0.70–0.81**)	**0.74 (0.69–0.79**)	**0.74 (0.69–0.80**)	**0.81 (0.75–0.86**)	**0.70 (0.65–0.76**)	**0.77 (0.67–0.88**)	**0.74 (0.65–0.84**)	**0.74 (0.66–0.83**)
	61–70	Low-level of household income	**2.04 (1.98–2.10**)	**1.80 (1.70–1.92**)	**1.83 (1.72–1.95**)	**1.81 (1.68–1.94**)	**1.89 (1.75–2.03**)	**1.86 (1.68–2.06**)	**1.80 (1.54–2.10**)	**2.06 (1.78–2.38**)	**2.07 (1.79–2.39**)
		Mid-level of household income	1.00 (ref)	1.00 (ref)	1.00 (ref)	1.00 (ref)	1.00 (ref)	1.00 (ref)	1.00 (ref)	1.00 (ref)	1.00 (ref)
		High-level of household income	**0.67 (0.63–0.71**)	**0.69 (0.59–0.81**)	**0.72 (0.62–0.83**)	**0.68 (0.56–0.81**)	0.88 (0.76–1.02)	**0.68 (0.58–0.78**)	**0.76 (0.60–0.95**)	0.82 (0.66–1.01)	**0.61 (0.51–0.75**)
	71–80	Low-level of household income	**2.18 (2.10–2.25**)	**1.95 (1.81–2.11**)	**1.85 (1.71–1.99**)	**1.81 (1.67–1.97**)	**2.01 (1.83–2.19**)	**2.34 (2.12–2.58**)	**1.78 (1.51–2.11**)	**1.76 (1.50–2.06**)	**1.81 (1.57–2.09**)
		Mid-level of household income	1.00 (ref)	1.00 (ref)	1.00 (ref)	1.00 (ref)	1.00 (ref)	1.00 (ref)	1.00 (ref)	1.00 (ref)	1.00 (ref)
		High-level of household income	**0.72 (0.65–0.79**)	**0.80 (0.65–0.99**)	**0.73 (0.60–0.90**)	0.83 (0.63–1.10)	**0.64 (0.50–0.84**)	**0.74 (0.55–0.98**)	0.66 (0.40–1.07)	**0.53 (0.31–0.90**)	**0.37 (0.24–0.57**)
	Over 81	Low-level of household income	**1.98 (1.87–2.09**)	**1.72 (1.51–1.96**)	**1.92 (1.68–2.19**)	**1.93 (1.68–2.21**)	**1.88 (1.65–2.14**)	**1.91 (1.67–2.19**)	**2.11 (1.65–2.71**)	**1.49 (1.21–1.84**)	**2.34 (1.95–2.82**)
		Mid-level of household income	1.00 (ref)	1.00 (ref)	1.00 (ref)	1.00 (ref)	1.00 (ref)	1.00 (ref)	1.00 (ref)	1.00 (ref)	1.00 (ref)
		High-level of household income	**0.68 (0.60–0.78**)	**0.51 (0.35–0.74**)	**0.67 (0.49–0.92**)	**0.57 (0.37–0.86**)	**0.63 (0.43–0.91**)	0.79 (0.57–1.09)	1.09 (0.62–1.91)	**0.54 (0.30–0.97**)	0.92 (0.59–1.43)
Sex	Male	Low-level of household income	**1.24 (1.22–1.27**)	**1.23 (1.17–1.28**)	**1.26 (1.20–1.33**)	**1.17 (1.11–1.22**)	**1.15 (1.09–1.21**)	**1.41 (1.30–1.52**)	**1.13 (0.99–1.29**)	**1.66 (1.47–1.88**)	**1.92 (1.72–2.14**)
		Mid-level of household income	1.00 (ref)	1.00 (ref)	1.00 (ref)	1.00 (ref)	1.00 (ref)	1.00 (ref)	1.00 (ref)	1.00 (ref)	1.00 (ref)
		High-level of household income	**0.79 (0.77–0.80**)	**0.86 (0.82–0.90**)	**0.86 (0.82–0.90**)	**0.89 (0.85–0.93**)	**0.91 (0.87–0.95**)	**0.88 (0.83–0.93**)	**0.82 (0.74–0.91**)	**0.85 (0.76–0.94**)	0.91 (0.83–1.00)
	Female	Low-level of household income	**1.31 (1.29–1.33**)	**1.34 (1.30–1.38**)	**1.39 (1.35–1.44**)	**1.27 (1.22–1.31**)	**1.22 (1.18–1.27**)	**1.36 (1.29–1.43**)	**1.22 (1.12–1.33**)	**1.37 (1.26–1.48**)	**1.47 (1.35–1.59**)
		Mid-level of household income	1.00 (ref)	1.00 (ref)	1.00 (ref)	1.00 (ref)	1.00 (ref)	1.00 (ref)	1.00 (ref)	1.00 (ref)	1.00 (ref)
		High-level of household income	**0.79 (0.78–0.81**)	**0.83 (0.80–0.87**)	**0.87 (0.84–0.91**)	**0.88 (0.85–0.92**)	**0.92 (0.89–0.96**)	**0.87 (0.83–0.92**)	**0.90 (0.83–0.98**)	**0.86 (0.79–0.93**)	**0.85 (0.79–0.92**)

BMI = body mass index; CI = confidence interval; KCHS = Korea Community Health Survey; OR = odds ratios.

The numbers in bold indicate significant differences (*P* <.05).

* According to the Asian–Pacific guidelines, the BMI is divided into 4 groups: underweight (<18.5 kg/m^2^), normal (18.5–22.9 kg/m^2^), overweight (23.0–24.9 kg/m^2^), and obese (≥25.0 kg/m^2^).

Table 4 also highlights unmet healthcare needs by education level. The disparity in unmet healthcare needs based on income is more pronounced among those with a high school or lower education compared to those with a college or higher education (ROR, high school or lower education: low-level of household income: 1.50 [95% CI, 1.47–1.53]; high-level of household income: 0.81 [95% CI, 0.78–0.85]; college or higher education: low-level of household income: 1.21 [95% CI, 1.18–1.23]; high-level of household income: 0.78 [95% CI, 0.76–0.79]). Similarly, it was observed that the disparity in unmet healthcare needs due to income differences was more pronounced among individuals with lower subjective health-levels (ROR, high health-level: low-level of household income: 0.98 [95% CI, 0.94–1.02]; high-level of household income: 0.80 [95% CI, 0.78–0.82]; middle health-level: low-level of household income: 0.93 [95% CI, 0.90–0.95]; high-level of household income: 0.85 [95% CI, 0.84–0.87]; low health level: low-level of household income: 1.25 [95% CI, 1.23–1.28]; high-level of household income: 0.92 [95% CI, 0.89–0.95]). These results highlight a significant gap in healthcare access based on income, particularly among individuals with lower educational attainment and lower subjective health.

## 4. Discussion

### 4.1. Main findings

The present study addresses a significant gap in understanding healthcare disparities across socio-economic groups using longitudinal data from a large representative sample (n = 2,628,584). The onset of the COVID-19 pandemic accelerated disparities in healthcare access, reversing the previous trend of declining unmet healthcare needs into increasing them, especially in low-income households. We consistently found higher rates of unmet healthcare needs among low-income households compared to their high-income households. In addition to income disparities, our study also identified significant differences in age, education level, and subjective health status. Older age groups in low-income households consistently showed significantly higher rates of unmet healthcare needs. Education also played a crucial role in healthcare disparities, as individuals with a high school or lower education experienced a greater disparity in unmet healthcare needs based on income compared to those with a college or higher education. Similarly, respondents with low subjective health levels exhibited a significant gap in unmet healthcare needs between high and low household income levels. These disparities persisted across all time intervals examined in the study, highlighting the pandemic’s disruptive effect on healthcare services, especially for vulnerable populations.

### 4.2. Interpretations of the study findings

The prevalence of unmet healthcare needs is considerably greater among low-income households than among high-income households. This may be attributed to the relatively high out-of-pocket share of 29 % under Korea’s national health insurance – one of the highest among Organization for Economic Co-operation and Development countries – and to the fact that patients incur 20 %–60 % (or more) of the costs for both outpatient and inpatient care.^[22,23]^ The study also highlights a notable increase in unmet healthcare needs during the COVID-19 pandemic, which contrasts with the previous trend of decreasing unmet needs observed from 2010 to 2020. A parallel trend of unmet healthcare needs has been observed in Istanbul and across Europe, with a significant increase during the COVID-19 pandemic.^[24,25]^ This phenomenon can be attributed to several factors. The pandemic triggered an unprecedented public health crisis that overwhelmed global healthcare systems. Hospitals and clinics had to prioritize patients with COVID-19, which limited their ability to address other healthcare needs, such as managing chronic diseases, conducting routine checkups, and performing surgeries, many of which were frequently postponed or canceled.^[26]^

Many people experienced income reductions and widespread job losses due to the pandemic’s economic downturn, particularly those from low-income households.^[27]^ Their incapacity to afford healthcare services and associated expenses, including transportation, prescription drugs, and health insurance, was a result of their financial challenges. This forced many people in low-income groups to delay essential medical care, worsening existing health inequities.^[28,29]^ Lockdowns, social distancing policies, and funding reductions posed significant operational issues for community health programs and nonprofits, which provide vital services to underserved communities. These disruptions hindered their ability to offer medical assistance, counseling, and other support.^[30]^

Additionally, funding cuts further worsened the situation. Public health programs and community-based organizations faced reduced budgets as government resources were diverted to address the immediate impacts of the pandemic. Nonprofits and charitable organizations, relying heavily on donations, saw a significant decline due to the economic downturn affecting donors. These financial constraints limited their ability to provide essential services such as medical assistance, mental health counseling, and social support, which are crucial for vulnerable populations.^[28]^

Social distancing measures, crucial for controlling the spread of the virus, deterred people from visiting hospitals and other crowded places. This is due to individuals’ concern about contracting the infection. Despite the comprehensive healthcare benefits available to the older population in South Korea,^[31]^ significant challenges persisted, especially for older adults in low-income groups. These individuals encountered additional barriers in accessing essential healthcare services. The higher average age within the low-income cohort indicates a link between older age and limited financial resources, exacerbating difficulties in accessing healthcare. The fear of exposure to SARS-CoV-2 in healthcare settings led to a decrease in preventive care and management of chronic conditions. Unmet healthcare needs were especially higher among older adults, who were more prone to severe COVID-19 complications. The heightened risk of severe illness and mortality from COVID-19 led many older individuals to avoid visiting hospitals and clinics, resulting in delayed diagnoses and treatment of other health issues.^[32]^

The study also found that higher-educated people typically see fewer healthcare inequities between low- and high-income households. This suggests that education plays a significant role in mitigating healthcare inequalities. Higher-educated individuals are more likely to hold steady jobs with insurance, which lowers the cost barriers to receiving medical treatment.^[33]^

Addressing these challenges requires targeted policy interventions that enhance the accessibility and affordability of healthcare services, particularly for older adults and those facing socio-economic disadvantage. By addressing these issues comprehensively, policymakers can promote healthcare equity in preparation for future challenges.

### 4.3. Comparison with previous studies

This study examined the determinants of unmet healthcare needs among Korean adults using KCHS data from 2010 to 2022. A previous study identified that younger adults, particularly those aged 19 to 39 years, face higher odds of experiencing unmet healthcare needs compared to older adults, regardless of sex.^[29]^ In contrast to previous research that primarily highlighted increased unmet healthcare requirements among younger persons, our data show a significant divergence. Our study reveals that older age groups within low-income households continuously had significantly higher rates of unmet healthcare needs. These unexpected findings challenge previous assumptions and highlight the complex interplay between access to healthcare, age, and income levels. It also suggests a more sophisticated view of healthcare disparities.

Moreover, previous studies typically focused on data from 2013 to 2017 or analyzed only the initial COVID-19 outbreak for 1 year and did not explore the potential impact of the COVID-19 pandemic on healthcare needs.^[7,9]^ In contrast, our study extends from 2010 to 2022 and explicitly analyzes healthcare disparities across socio-economic groups, with a specific emphasis on the pandemic’s effects. Before the onset of the pandemic, our study also observed a positive trend where unmet healthcare needs were declining across all income groups. However, with the outbreak of COVID-19, this promising trend reversed sharply, resulting in a significant increase in the rate of unmet healthcare needs across the board. This crisis has disproportionately affected low-income households, exacerbated their existing challenges, and underscored the critical role of external factors, such as economic conditions and public health emergencies, in shaping disparities in healthcare access. Understanding these dynamics is crucial for developing effective strategies and ensuring more equitable access to healthcare in the future.

Additionally, our study identifies educational attainment and subjective health status as critical factors influencing disparities in healthcare access. These insights highlight how broader socio-economic factors interact with individual health perceptions to shape disparities in healthcare access. By uncovering these layers of influence, our research provides a clearer understanding of healthcare disparities. It underscores the urgent need for targeted interventions and policies to ensure equitable access to healthcare across diverse socio-economic backgrounds.

### 4.4. Policy implication

Addressing the growing issue of unmet healthcare needs, particularly among older individuals in low-income groups, is crucial for policymakers and healthcare professionals.^[34]^ This study underscores the vulnerability of these demographics to healthcare gaps during the COVID-19 pandemic. To combat this, targeted interventions are necessary.

There is an urgent need to prioritize public health campaigns that promote preventive healthcare behaviors, especially among low-income households. Simultaneously, there is a need to improve awareness among policymakers and healthcare practitioners about the significant impact of socio-economic status on unmet healthcare needs. Advocating for more funding and resources is critical to advancing research and programs that address these disparities.^[30,35]^

Financial barriers often hinder access to healthcare for many individuals. To alleviate these challenges, targeted financial support and enhancements in insurance coverage are imperative. Initiatives such as expanding Medicaid coverage, subsidizing health insurance premiums, and implementing income-based sliding scales for healthcare costs can enhance affordability and reduce disparities in accessing necessary medical services.^[36]^

Addressing social determinants of health is crucial for promoting equitable access to healthcare. Initiatives to improve employment opportunities are essential for mitigating socio-economic disparities and enhancing health outcomes across diverse populations. It is equally essential to develop tailored strategies for groups with low education and low subjective health, who experience relatively severe disparities in unmet healthcare needs due to income inequality. These strategies should prioritize improving access to affordable healthcare, enhancing health literacy, and removing barriers to individuals seeking necessary medical care.^[28,33]^ By implementing these measures, policymakers and healthcare professionals can effectively reduce unmet healthcare needs and advance health equity among vulnerable populations.

## 5. Strengths and limitations

This study effectively highlights trends in unmet healthcare needs based on household income levels using 12 years of nationally representative, large-scale data. It represents the most current and comprehensive analysis of unmet healthcare needs, including data from both before and during the COVID-19 pandemic. This robust approach allows us to identify and understand different risk factors associated with socio-economic status and age, incorporating variables such as sex, education level, BMI, and smoking habits. By including all adult age groups, our study uniquely identifies significant trends in unmet healthcare needs across adulthood. The findings provide crucial insights for health policymakers and professionals, enabling the development of targeted, income-specific health interventions to address unmet healthcare needs and reduce long-term socio-economic costs.

However, this study has some limitations. First, the dataset is self-reported, which may introduce several types of reporting bias.^[37]^ Recall bias may occur if participants fail to accurately remember past healthcare experiences, such as the timing or frequency of unmet medical needs.^[37]^ Information bias could arise from misreporting or misunderstanding survey questions, leading to inaccurate or inconsistent responses.^[38]^ Selection bias may also be present if individuals with poorer health status or limited healthcare access were less likely to participate in the survey.^[38]^ Collectively, these biases could contribute to an underestimation of the true prevalence of unmet healthcare needs.^[39]^ Second, the data is limited to Koreans, which may restrict the generalizability of the findings to other populations. Therefore, further investigation is necessary to confirm these results on a global scale and to examine the root causes and consequences of unmet healthcare needs in diverse socio-economic contexts.^[40]^ Third, the unmet healthcare needs assessed in this study may have been influenced by a range of contextual and structural factors beyond those directly examined. Although we primarily emphasized the role of financial constraints, additional determinants such as occupational conditions, environmental exposures, and public health infrastructure may also shape healthcare accessibility.^[41]^ For instance, long or irregular working hours, shift-based employment, or lack of paid sick leave can limit opportunities to seek medical care despite available resources. Similarly, environmental factors such as geographic disparities, transportation barriers, or regional shortages of healthcare resources may further hinder access to services.^[42]^ Moreover, changes in public health policies, healthcare delivery systems, and community-level interventions during and after the pandemic could have also influenced individuals’ perceived and actual unmet healthcare needs.^[9]^ Therefore, these broader contextual elements should be considered when interpreting our findings and assessing the generalizability of the results. Despite these limitations, the study provides valuable, country-specific data on unmet healthcare needs that can inform future research and policy-making, acknowledging that characteristics of unmet needs may differ across nations.

## 6. Conclusions

Based on the comprehensive analysis of unmet healthcare needs among Korean adults from 2010 to 2022, this study provides important insights into the determinants and trends exacerbated by the COVID-19 pandemic. Before the pandemic, unmet healthcare needs were declining across all income groups. However, the emergence of COVID-19 reversed this trend, particularly exacerbating disparities among vulnerable populations, such as low-income households. Economic downturns reduced access to healthcare, and heightened public health concerns during the pandemic exacerbated these disparities. In response to these findings, targeted interventions are urgently needed. Efforts should prioritize public health campaigns to promote preventive care and raise awareness of the impact of socio-economic status on healthcare access. Enhanced funding, financial support, and improved insurance coverage can help mitigate financial barriers and increase affordability. These measures can address the study’s challenges, promoting equitable access to healthcare and improved health outcomes.

## Author contributions

**Conceptualization:** Hyunjee Kim, Jaeyu Park, Jinyoung Jeong, Saiah Kim, Lee Smith, Dong Keon Yon.

**Data curation:** Hyunjee Kim, Jaeyu Park, Jinyoung Jeong, Saiah Kim, Lee Smith, Dong Keon Yon.

**Formal analysis:** Hyunjee Kim, Jaeyu Park, Jinyoung Jeong, Saiah Kim, Lee Smith, Dong Keon Yon.

**Funding acquisition:** Lee Smith, Dong Keon Yon.

**Investigation:** Hyunjee Kim, Jaeyu Park, Jinyoung Jeong, Saiah Kim, Lee Smith, Dong Keon Yon.

**Methodology:** Hyunjee Kim, Jaeyu Park, Jinyoung Jeong, Saiah Kim, Lee Smith, Dong Keon Yon.

**Project administration:** Hyunjee Kim, Jaeyu Park, Jinyoung Jeong, Saiah Kim, Lee Smith, Dong Keon Yon.

**Resources:** Hyunjee Kim, Jaeyu Park, Jinyoung Jeong, Saiah Kim, Lee Smith, Dong Keon Yon.

**Software:** Hyunjee Kim, Jaeyu Park, Jinyoung Jeong, Saiah Kim, Lee Smith, Dong Keon Yon.

**Supervision:** Ho Geol Woo, Dong Keon Yon.

**Validation:** Hyunjee Kim, Jaeyu Park, Jinyoung Jeong, Saiah Kim, Lee Smith, Dong Keon Yon.

**Visualization:** Hyunjee Kim, Jaeyu Park, Jinyoung Jeong, Saiah Kim, Lee Smith, Dong Keon Yon.

**Writing – original draft:** Hyunjee Kim, Jaeyu Park, Jinyoung Jeong, Saiah Kim, Lee Smith, Dong Keon Yon.

**Writing – review & editing:** Hyunjee Kim, Jaeyu Park, Jinyoung Jeong, Saiah Kim, Hayeon Lee, Hyeon Jin Kim, Yejun Son, Soeun Kim, Sooji Lee, Kyeongmin Lee, Hyesu Jo, Yesol Yim, Masoud Rahmati, Damiano Pizzol, Lee Smith, Dong Keon Yon.

## Supplementary Material


