# Supplementary material for: National trends of unmet healthcare needs and risk factors by household income level, 2010 to 2022: A Nationwide cross-sectional study in South Korea
Source: Medicine (Baltimore). 2026 Jan 23;105(4):e47143. doi: 10.1097/MD.0000000000047143 (PMC12851669; doi:10.1097/MD.0000000000047143)
Supplement: Supplementary file 1 [file medi-105-e47143-s001.docx]

**Table S1.** National trends in the prevalence of unmet healthcare needs, before and during the COVID-19 pandemic, presented as weighted percentages with 95% confidence intervals, using data obtained from KCHS.

| Variables | | | Pre-pandemic | | | | | | | | | | Pandemic | | |
| --- | --- | --- | --- | --- | --- | --- | --- | --- | --- | --- | --- | --- | --- | --- | --- |
|  |  |  | 2010 | 2011 | 2012 | 2013 | 2014 | 2015 | 2016 | 2017 | 2018 | 2019 | 2020 | 2021 | 2022 |
| Overall | | Low-level of household income | 21.23 (20.69 to 21.77) | 19.63 (19.06 to 20.21) | 17.01 (16.45 to 17.56) | 18.37 (17.80 to 18.95) | 14.86 (14.39 to 15.32) | 14.52 (14.04 to 15.00) | 13.92 (13.46 to 14.37) | 13.03 (12.57 to 13.50) | 12.22 (11.66 to 12.78) | 8.91 (8.40 to 9.41) | 6.51 (6.09 to 6.94) | 7.75 (7.28 to 8.23) | 8.79 (8.29 to 9.29) |
|  |  | Mid-level of household income | 17.06 (16.79 to 17.33) | 13.73 (13.48 to 13.98) | 12.05 (11.82 to 12.28) | 13.54 (13.29 to 13.79) | 11.57 (11.35 to 11.79) | 11.70 (11.48 to 11.93) | 11.43 (11.21 to 11.65) | 10.56 (10.34 to 10.77) | 8.87 (8.64 to 9.10) | 6.06 (5.86 to 6.27) | 5.34 (5.15 to 5.54) | 5.17 (4.98 to 5.36) | 5.31 (5.12 to 5.50) |
|  |  | High-level of household income | 14.74 (14.23 to 15.25) | 11.46 (11.05 to 11.88) | 10.23 (9.87 to 10.60) | 11.84 (11.46 to 12.23) | 10.21 (9.82 to 10.60) | 10.36 (9.96 to 10.75) | 10.53 (10.15 to 10.90) | 9.66 (9.32 to 10.01) | 7.84 (7.54 to 8.13) | 5.25 (4.99 to 5.52) | 4.62 (4.36 to 4.87) | 4.39 (4.15 to 4.63) | 4.64 (4.41 to 4.88) |
| Age, years | 19-30 | Low-level of household income | 21.11 (19.14 to 23.08) | 14.72 (12.44 to 17.01) | 12.96 (10.82 to 15.11) | 13.64 (11.60 to 15.68) | 14.49 (12.74 to 16.24) | 14.39 (12.51 to 16.27) | 13.97 (12.18 to 15.75) | 12.62 (10.78 to 14.47) | 11.69 (9.48 to 13.90) | 6.80 (4.73 to 8.88) | 7.45 (5.21 to 9.70) | 8.90 (6.41 to 11.39) | 7.18 (4.78 to 9.58) |
|  |  | Mid-level of household income | 18.78 (18.21 to 19.36) | 13.31 (12.76 to 13.85) | 12.56 (12.01 to 13.11) | 13.42 (12.84 to 14.00) | 12.82 (12.30 to 13.35) | 13.09 (12.55 to 13.63) | 12.74 (12.18 to 13.29) | 11.94 (11.37 to 12.51) | 10.40 (9.77 to 11.03) | 6.93 (6.37 to 7.50) | 6.20 (5.65 to 6.75) | 5.69 (5.14 to 6.25) | 6.35 (5.74 to 6.96) |
|  |  | High-level of household income | 16.45 (15.35 to 17.54) | 11.28 (10.38 to 12.18) | 10.99 (10.17 to 11.81) | 11.88 (11.06 to 12.70) | 11.87 (10.98 to 12.76) | 11.77 (10.88 to 12.67) | 11.23 (10.43 to 12.02) | 10.83 (10.08 to 11.59) | 7.59 (7.00 to 8.19) | 5.86 (5.27 to 6.45) | 4.54 (4.01 to 5.08) | 3.93 (3.42 to 4.43) | 4.72 (4.18 to 5.27) |
|  | 31-40 | Low-level of household income | 23.81 (21.39 to 26.23) | 17.77 (15.37 to 20.16) | 16.68 (13.94 to 19.43) | 21.90 (18.39 to 25.41) | 17.16 (14.56 to 19.76) | 18.52 (15.77 to 21.27) | 17.25 (14.37 to 20.14) | 16.99 (13.75 to 20.23) | 11.27 (7.57 to 14.98) | 11.14 (7.08 to 15.21) | 10.47 (6.18 to 14.77) | 9.92 (6.67 to 13.18) | 16.64 (11.22 to 22.06) |
|  |  | Mid-level of household income | 20.19 (19.65 to 20.74) | 15.81 (15.29 to 16.32) | 14.18 (13.67 to 14.68) | 15.86 (15.32 to 16.39) | 14.52 (14.01 to 15.02) | 14.39 (13.87 to 14.90) | 15.02 (14.48 to 15.55) | 13.35 (12.82 to 13.87) | 11.57 (10.95 to 12.19) | 7.31 (6.75 to 7.86) | 7.05 (6.48 to 7.62) | 6.19 (5.63 to 6.75) | 6.16 (5.61 to 6.72) |
|  |  | High-level of household income | 18.73 (17.63 to 19.83) | 13.43 (12.55 to 14.30) | 11.80 (11.03 to 12.56) | 14.84 (14.02 to 15.67) | 13.59 (12.64 to 14.55) | 13.34 (12.46 to 14.23) | 13.95 (13.04 to 14.86) | 12.50 (11.65 to 13.35) | 10.06 (9.29 to 10.83) | 6.44 (5.82 to 7.07) | 5.93 (5.25 to 6.60) | 4.82 (4.24 to 5.39) | 5.21 (4.66 to 5.76) |
|  | 41-50 | Low-level of household income | 25.80 (23.69 to 27.92) | 23.25 (21.13 to 25.38) | 20.20 (17.93 to 22.47) | 23.60 (21.11 to 26.08) | 18.76 (16.77 to 20.76) | 20.26 (17.99 to 22.54) | 17.74 (15.61 to 19.87) | 19.03 (16.61 to 21.45) | 15.43 (12.40 to 18.47) | 12.65 (9.32 to 15.99) | 9.58 (6.95 to 12.21) | 10.71 (8.14 to 13.29) | 15.90 (12.52 to 19.28) |
|  |  | Mid-level of household income | 18.21 (17.67 to 18.75) | 14.68 (14.17 to 15.19) | 12.41 (11.92 to 12.89) | 14.90 (14.37 to 15.42) | 11.67 (11.23 to 12.12) | 12.63 (12.16 to 13.10) | 12.29 (11.82 to 12.75) | 11.81 (11.32 to 12.30) | 9.95 (9.40 to 10.51) | 6.87 (6.34 to 7.39) | 6.70 (6.16 to 7.23) | 5.83 (5.33 to 6.33) | 5.94 (5.42 to 6.47) |
|  |  | High-level of household income | 14.36 (13.55 to 15.16) | 12.15 (11.40 to 12.90) | 11.08 (10.39 to 11.76) | 11.82 (11.16 to 12.48) | 9.93 (9.27 to 10.60) | 10.31 (9.59 to 11.02) | 10.92 (10.23 to 11.62) | 9.74 (9.09 to 10.39) | 8.91 (8.34 to 9.48) | 5.59 (5.08 to 6.10) | 4.94 (4.45 to 5.42) | 5.19 (4.68 to 5.69) | 5.29 (4.80 to 5.77) |
|  | 51-60 | Low-level of household income | 24.96 (23.47 to 26.46) | 23.57 (21.95 to 25.20) | 20.59 (18.96 to 22.23) | 22.25 (20.42 to 24.09) | 18.55 (17.21 to 19.89) | 18.84 (17.37 to 20.30) | 17.96 (16.50 to 19.41) | 16.45 (14.93 to 17.97) | 17.14 (14.63 to 19.64) | 13.60 (11.44 to 15.75) | 7.89 (6.48 to 9.30) | 11.92 (9.88 to 13.96) | 13.60 (11.50 to 15.70) |
|  |  | Mid-level of household income | 13.89 (13.37 to 14.40) | 13.19 (12.64 to 13.74) | 11.42 (10.94 to 11.90) | 13.19 (12.67 to 13.71) | 10.48 (10.03 to 10.93) | 10.37 (9.93 to 10.82) | 10.11 (9.68 to 10.53) | 9.73 (9.30 to 10.16) | 8.61 (8.13 to 9.09) | 6.69 (6.24 to 7.15) | 5.51 (5.09 to 5.93) | 5.94 (5.48 to 6.40) | 6.42 (5.94 to 6.89) |
|  |  | High-level of household income | 10.83 (9.88 to 11.78) | 9.65 (8.82 to 10.48) | 7.86 (7.16 to 8.56) | 10.15 (9.41 to 10.90) | 7.45 (6.73 to 8.17) | 8.02 (7.34 to 8.69) | 8.29 (7.60 to 8.98) | 7.74 (7.13 to 8.34) | 6.39 (5.87 to 6.91) | 4.47 (4.02 to 4.92) | 4.23 (3.77 to 4.68) | 4.39 (3.93 to 4.85) | 4.74 (4.31 to 5.17) |
|  | 61-70 | Low-level of household income | 19.21 (18.38 to 20.04) | 18.88 (17.92 to 19.83) | 15.86 (14.91 to 16.80) | 16.98 (15.99 to 17.96) | 13.00 (12.17 to 13.83) | 12.28 (11.44 to 13.11) | 12.26 (11.37 to 13.15) | 12.09 (11.22 to 12.96) | 11.45 (10.36 to 12.54) | 8.17 (7.17 to 9.17) | 6.69 (5.84 to 7.54) | 8.45 (7.42 to 9.47) | 8.72 (7.64 to 9.81) |
|  |  | Mid-level of household income | 10.48 (9.93 to 11.04) | 10.63 (10.03 to 11.23) | 8.67 (8.15 to 9.19) | 9.23 (8.72 to 9.74) | 6.85 (6.43 to 7.28) | 7.16 (6.71 to 7.61) | 6.81 (6.35 to 7.26) | 6.11 (5.73 to 6.49) | 6.01 (5.62 to 6.40) | 4.66 (4.28 to 5.03) | 3.72 (3.40 to 4.04) | 4.11 (3.78 to 4.45) | 4.22 (3.90 to 4.53) |
|  |  | High-level of household income | 7.51 (6.12 to 8.91) | 7.06 (5.76 to 8.35) | 5.39 (4.35 to 6.44) | 7.35 (6.15 to 8.54) | 3.99 (3.01 to 4.97) | 5.43 (4.21 to 6.65) | 5.92 (4.85 to 7.00) | 5.42 (4.38 to 6.46) | 4.55 (3.78 to 5.31) | 2.77 (2.26 to 3.29) | 2.81 (2.21 to 3.40) | 3.35 (2.71 to 4.00) | 2.59 (2.13 to 3.04) |
|  | 71-80 | Low-level of household income | 19.15 (18.38 to 19.91) | 18.84 (17.97 to 19.71) | 16.54 (15.64 to 17.44) | 17.09 (16.29 to 17.90) | 13.06 (12.37 to 13.75) | 11.93 (11.27 to 12.60) | 12.14 (11.46 to 12.82) | 10.83 (10.17 to 11.48) | 10.92 (10.18 to 11.66) | 7.61 (6.89 to 8.33) | 5.01 (4.48 to 5.54) | 5.58 (5.00 to 6.16) | 6.40 (5.78 to 7.02) |
|  |  | Mid-level of household income | 10.24 (9.51 to 10.96) | 9.28 (8.51 to 10.04) | 8.34 (7.63 to 9.05) | 9.80 (9.09 to 10.50) | 7.15 (6.49 to 7.80) | 6.68 (6.08 to 7.27) | 5.97 (5.36 to 6.58) | 5.49 (4.96 to 6.02) | 4.71 (4.27 to 5.14) | 3.35 (2.96 to 3.73) | 2.81 (2.44 to 3.17) | 3.18 (2.80 to 3.55) | 3.53 (3.17 to 3.90) |
|  |  | High-level of household income | 7.40 (5.64 to 9.16) | 8.22 (6.25 to 10.18) | 5.65 (3.99 to 7.31) | 7.43 (5.74 to 9.12) | 5.54 (3.62 to 7.47) | 5.94 (3.73 to 8.15) | 3.53 (2.36 to 4.70) | 3.84 (2.70 to 4.98) | 2.85 (1.86 to 3.85) | 3.04 (1.84 to 4.24) | 1.84 (1.03 to 2.65) | 1.67 (0.87 to 2.48) | 1.30 (0.77 to 1.83) |
|  | Over 81 | Low-level of household income | 21.75 (20.32 to 23.18) | 21.70 (20.08 to 23.32) | 17.88 (16.41 to 19.36) | 20.46 (18.94 to 21.98) | 16.54 (15.24 to 17.83) | 16.87 (15.49 to 18.25) | 15.11 (13.92 to 16.30) | 14.20 (13.11 to 15.29) | 12.57 (11.54 to 13.59) | 9.64 (8.67 to 10.62) | 6.66 (5.84 to 7.47) | 7.24 (6.41 to 8.06) | 8.69 (7.86 to 9.53) |
|  |  | Mid-level of household income | 13.92 (12.17 to 15.67) | 11.38 (9.68 to 13.07) | 9.81 (8.24 to 11.38) | 10.25 (8.97 to 11.52) | 8.44 (7.03 to 9.85) | 8.84 (7.47 to 10.21) | 7.16 (6.12 to 8.21) | 8.38 (7.16 to 9.60) | 6.80 (5.80 to 7.80) | 4.90 (4.10 to 5.70) | 3.15 (2.48 to 3.83) | 4.86 (4.01 to 5.70) | 3.71 (3.13 to 4.29) |
|  |  | High-level of household income | 6.25 (3.67 to 8.84) | 6.66 (3.81 to 9.50) | 6.06 (3.78 to 8.34) | 7.26 (4.65 to 9.87) | 6.16 (3.98 to 8.35) | 3.81 (1.64 to 5.97) | 5.34 (2.69 to 7.99) | 4.52 (2.59 to 6.45) | 6.26 (4.13 to 8.39) | 3.15 (1.76 to 4.54) | 3.43 (1.79 to 5.07) | 2.63 (1.30 to 3.96) | 3.41 (2.09 to 4.74) |
| Sex | Male | Low-level of household income | 17.94 (17.17 to 18.70) | 15.74 (14.93 to 16.56) | 13.38 (12.58 to 14.18) | 14.80 (13.95 to 15.64) | 11.85 (11.21 to 12.49) | 11.59 (10.93 to 12.26) | 11.40 (10.73 to 12.07) | 10.89 (10.22 to 11.56) | 10.26 (9.42 to 11.10) | 8.14 (7.33 to 8.94) | 5.28 (4.66 to 5.91) | 7.26 (6.45 to 8.07) | 8.65 (7.84 to 9.47) |
|  |  | Mid-level of household income | 15.18 (14.84 to 15.51) | 12.31 (11.98 to 12.64) | 10.35 (10.05 to 10.66) | 11.96 (11.64 to 12.29) | 9.93 (9.64 to 10.21) | 10.21 (9.92 to 10.50) | 9.96 (9.68 to 10.25) | 9.40 (9.11 to 9.68) | 7.72 (7.42 to 8.03) | 5.26 (4.99 to 5.53) | 4.67 (4.41 to 4.93) | 4.37 (4.13 to 4.62) | 4.51 (4.26 to 4.76) |
|  |  | High-level of household income | 13.14 (12.50 to 13.78) | 10.60 (10.05 to 11.14) | 8.95 (8.47 to 9.43) | 10.11 (9.62 to 10.60) | 8.92 (8.42 to 9.42) | 8.99 (8.48 to 9.50) | 9.29 (8.81 to 9.76) | 8.34 (7.90 to 8.79) | 6.89 (6.51 to 7.28) | 4.60 (4.27 to 4.94) | 3.83 (3.49 to 4.16) | 3.70 (3.38 to 4.02) | 4.12 (3.82 to 4.42) |
|  | Female | Low-level of household income | 23.52 (22.87 to 24.17) | 22.31 (21.60 to 23.01) | 19.47 (18.78 to 20.16) | 20.82 (20.13 to 21.52) | 17.01 (16.42 to 17.60) | 16.55 (15.93 to 17.17) | 15.63 (15.06 to 16.21) | 14.51 (13.92 to 15.11) | 13.47 (12.81 to 14.13) | 9.38 (8.77 to 9.99) | 7.29 (6.77 to 7.82) | 8.07 (7.52 to 8.62) | 8.88 (8.28 to 9.48) |
|  |  | Mid-level of household income | 19.00 (18.64 to 19.35) | 15.20 (14.86 to 15.54) | 13.79 (13.47 to 14.11) | 15.16 (14.82 to 15.50) | 13.27 (12.96 to 13.58) | 13.26 (12.95 to 13.56) | 12.94 (12.63 to 13.25) | 11.74 (11.44 to 12.04) | 10.00 (9.68 to 10.32) | 6.81 (6.53 to 7.10) | 5.98 (5.72 to 6.25) | 5.91 (5.64 to 6.19) | 6.05 (5.79 to 6.32) |
|  |  | High-level of household income | 16.40 (15.72 to 17.07) | 12.36 (11.80 to 12.92) | 11.55 (11.04 to 12.07) | 13.61 (13.09 to 14.14) | 11.54 (10.98 to 12.09) | 11.75 (11.19 to 12.30) | 11.80 (11.27 to 12.33) | 11.03 (10.53 to 11.52) | 8.80 (8.38 to 9.22) | 5.91 (5.55 to 6.27) | 5.40 (5.03 to 5.76) | 5.06 (4.71 to 5.40) | 5.15 (4.84 to 5.47) |
| Residential area | Urban | Low-level of household income | 23.00 (22.17 to 23.82) | 20.46 (19.63 to 21.30) | 17.25 (16.42 to 18.08) | 18.25 (17.42 to 19.09) | 14.75 (14.11 to 15.39) | 14.45 (13.79 to 15.12) | 13.85 (13.21 to 14.49) | 13.05 (12.40 to 13.70) | 12.30 (11.50 to 13.11) | 8.92 (8.20 to 9.63) | 6.13 (5.56 to 6.70) | 7.72 (7.10 to 8.34) | 8.91 (8.24 to 9.57) |
|  |  | Mid-level of household income | 17.70 (17.38 to 18.02) | 13.75 (13.46 to 14.04) | 12.27 (12.00 to 12.53) | 13.69 (13.40 to 13.98) | 11.69 (11.44 to 11.95) | 11.65 (11.40 to 11.91) | 11.47 (11.22 to 11.73) | 10.65 (10.40 to 10.91) | 8.83 (8.57 to 9.09) | 6.09 (5.86 to 6.33) | 5.31 (5.09 to 5.54) | 5.13 (4.91 to 5.35) | 5.22 (5.01 to 5.44) |
|  |  | High-level of household income | 14.99 (14.42 to 15.57) | 11.18 (10.74 to 11.63) | 10.34 (9.94 to 10.74) | 11.94 (11.52 to 12.37) | 10.29 (9.86 to 10.72) | 9.96 (9.55 to 10.37) | 10.44 (10.03 to 10.85) | 9.62 (9.24 to 10.00) | 7.79 (7.47 to 8.11) | 5.28 (4.99 to 5.57) | 4.47 (4.19 to 4.74) | 4.27 (4.01 to 4.53) | 4.53 (4.28 to 4.78) |
|  | Rural | Low-level of household income | 18.31 (17.88 to 18.74) | 18.28 (17.65 to 18.91) | 16.63 (16.04 to 17.21) | 18.57 (17.94 to 19.20) | 15.07 (14.50 to 15.64) | 14.65 (14.04 to 15.25) | 14.04 (13.49 to 14.59) | 13.00 (12.42 to 13.57) | 12.07 (11.45 to 12.68) | 8.88 (8.29 to 9.48) | 7.29 (6.72 to 7.86) | 7.84 (7.21 to 8.48) | 8.52 (7.88 to 9.17) |
|  |  | Mid-level of household income | 14.28 (13.90 to 14.66) | 13.62 (13.17 to 14.08) | 11.12 (10.71 to 11.53) | 12.89 (12.47 to 13.32) | 11.01 (10.60 to 11.43) | 11.93 (11.51 to 12.35) | 11.26 (10.87 to 11.65) | 10.15 (9.76 to 10.53) | 9.03 (8.56 to 9.51) | 5.93 (5.59 to 6.28) | 5.47 (5.11 to 5.83) | 5.33 (4.96 to 5.69) | 5.70 (5.33 to 6.06) |
|  |  | High-level of household income | 12.93 (12.23 to 13.63) | 13.69 (12.56 to 14.81) | 9.37 (8.60 to 10.13) | 11.08 (10.25 to 11.91) | 9.57 (8.60 to 10.55) | 13.66 (12.28 to 15.05) | 11.22 (10.36 to 12.07) | 9.98 (9.18 to 10.77) | 8.22 (7.43 to 9.00) | 5.03 (4.50 to 5.57) | 5.69 (4.97 to 6.41) | 5.24 (4.61 to 5.87) | 5.41 (4.82 to 5.99) |
| BMI^*^ | Underweight | Low-level of household income | 25.47 (24.26 to 26.67) | 22.60 (21.37 to 23.82) | 21.12 (19.94 to 22.30) | 22.03 (20.87 to 23.19) | 18.48 (17.25 to 19.71) | 18.68 (17.38 to 19.98) | 16.63 (15.54 to 17.73) | 16.25 (14.94 to 17.56) | 13.78 (12.70 to 14.85) | 14.10 (12.40 to 15.80) | 7.81 (6.04 to 9.59) | 9.20 (7.66 to 10.73) | 11.34 (9.71 to 12.97) |
|  |  | Mid-level of household income | 19.82 (18.80 to 20.84) | 14.87 (13.96 to 15.78) | 14.21 (13.29 to 15.14) | 15.83 (14.89 to 16.78) | 13.41 (12.54 to 14.28) | 13.09 (12.21 to 13.97) | 13.09 (12.15 to 14.03) | 12.19 (11.26 to 13.12) | 9.34 (8.49 to 10.20) | 7.28 (6.51 to 8.05) | 7.39 (6.28 to 8.49) | 6.47 (5.49 to 7.45) | 5.82 (4.95 to 6.70) |
|  |  | High-level of household income | 16.05 (14.22 to 17.87) | 12.53 (10.94 to 14.12) | 12.01 (10.51 to 13.51) | 14.12 (12.52 to 15.72) | 10.76 (9.17 to 12.34) | 12.16 (10.42 to 13.89) | 13.21 (11.39 to 15.02) | 10.83 (9.24 to 12.41) | 8.60 (7.28 to 9.93) | 5.30 (4.42 to 6.18) | 5.12 (3.85 to 6.39) | 5.02 (3.88 to 6.16) | 5.26 (4.16 to 6.35) |
|  | Normal weight | Low-level of household income | 20.67 (19.89 to 21.46) | 19.61 (18.74 to 20.48) | 16.23 (15.42 to 17.04) | 17.38 (16.52 to 18.24) | 14.82 (14.11 to 15.54) | 14.08 (13.39 to 14.77) | 13.99 (13.26 to 14.72) | 12.75 (12.06 to 13.45) | 13.05 (12.06 to 14.05) | 8.15 (7.33 to 8.96) | 6.70 (6.04 to 7.35) | 7.48 (6.77 to 8.18) | 8.51 (7.74 to 9.28) |
|  |  | Mid-level of household income | 17.38 (17.00 to 17.76) | 13.79 (13.45 to 14.14) | 12.25 (11.91 to 12.59) | 13.57 (13.21 to 13.92) | 11.80 (11.48 to 12.12) | 12.02 (11.69 to 12.35) | 11.65 (11.32 to 11.98) | 10.70 (10.37 to 11.03) | 9.05 (8.68 to 9.41) | 6.07 (5.74 to 6.40) | 5.21 (4.91 to 5.51) | 5.28 (4.98 to 5.59) | 5.36 (5.06 to 5.65) |
|  |  | High-level of household income | 15.07 (14.38 to 15.76) | 11.53 (10.95 to 12.10) | 10.45 (9.93 to 10.98) | 11.90 (11.37 to 12.42) | 10.62 (10.06 to 11.18) | 10.79 (10.21 to 11.36) | 10.48 (9.95 to 11.02) | 9.82 (9.33 to 10.31) | 7.72 (7.28 to 8.16) | 5.32 (4.93 to 5.71) | 4.72 (4.34 to 5.10) | 4.17 (3.80 to 4.53) | 4.69 (4.35 to 5.04) |
|  | Overweight | Low-level of household income | 19.78 (18.71 to 20.85) | 17.08 (15.93 to 18.22) | 14.36 (13.29 to 15.42) | 17.05 (15.87 to 18.23) | 13.79 (12.84 to 14.74) | 12.85 (11.92 to 13.77) | 12.33 (11.46 to 13.20) | 11.91 (10.98 to 12.84) | 9.81 (8.76 to 10.85) | 8.69 (7.55 to 9.83) | 5.85 (5.03 to 6.66) | 6.53 (5.61 to 7.44) | 7.76 (6.85 to 8.67) |
|  |  | Mid-level of household income | 15.76 (15.29 to 16.23) | 12.96 (12.49 to 13.43) | 11.14 (10.70 to 11.57) | 12.97 (12.51 to 13.42) | 10.73 (10.31 to 11.15) | 10.62 (10.22 to 11.03) | 10.70 (10.27 to 11.12) | 9.73 (9.33 to 10.14) | 8.29 (7.86 to 8.72) | 5.76 (5.36 to 6.15) | 4.96 (4.60 to 5.32) | 4.73 (4.38 to 5.08) | 5.12 (4.76 to 5.49) |
|  |  | High-level of household income | 14.28 (13.36 to 15.19) | 10.52 (9.75 to 11.30) | 9.23 (8.55 to 9.92) | 11.28 (10.57 to 12.00) | 9.63 (8.86 to 10.40) | 9.94 (9.20 to 10.68) | 9.92 (9.20 to 10.63) | 9.20 (8.52 to 9.88) | 7.26 (6.70 to 7.83) | 4.83 (4.30 to 5.35) | 4.34 (3.87 to 4.81) | 4.70 (4.20 to 5.21) | 4.75 (4.27 to 5.22) |
|  | Obese | Low-level of household income | 21.03 (19.93 to 22.14) | 20.07 (18.90 to 21.24) | 18.02 (16.72 to 19.31) | 18.57 (17.35 to 19.79) | 13.81 (12.94 to 14.68) | 14.43 (13.44 to 15.41) | 13.79 (12.88 to 14.70) | 12.99 (12.05 to 13.93) | 11.97 (10.93 to 13.02) | 8.14 (7.35 to 8.93) | 6.55 (5.83 to 7.27) | 8.80 (7.82 to 9.78) | 9.26 (8.29 to 10.23) |
|  |  | Mid-level of household income | 17.00 (16.51 to 17.48) | 14.06 (13.57 to 14.54) | 11.98 (11.53 to 12.43) | 13.46 (13.00 to 13.93) | 11.52 (11.11 to 11.93) | 11.86 (11.44 to 12.28) | 11.41 (11.02 to 11.80) | 10.74 (10.35 to 11.14) | 9.00 (8.61 to 9.39) | 5.98 (5.64 to 6.31) | 5.55 (5.22 to 5.89) | 5.18 (4.86 to 5.51) | 5.32 (5.00 to 5.65) |
|  |  | High-level of household income | 14.13 (13.17 to 15.09) | 12.00 (11.14 to 12.86) | 10.30 (9.59 to 11.01) | 11.71 (11.00 to 12.43) | 9.81 (9.07 to 10.56) | 9.54 (8.80 to 10.27) | 10.61 (9.90 to 11.31) | 9.58 (8.94 to 10.21) | 8.34 (7.81 to 8.86) | 5.47 (5.02 to 5.92) | 4.62 (4.17 to 5.06) | 4.36 (3.94 to 4.78) | 4.40 (4.04 to 4.77) |
| Education | High school or lower education | Low-level of household income | 21.12 (20.56 to 21.67) | 21.08 (20.44 to 21.72) | 18.01 (17.37 to 18.65) | 19.27 (18.65 to 19.89) | 15.39 (14.85 to 15.92) | 14.94 (14.39 to 15.50) | 14.38 (13.84 to 14.92) | 13.22 (12.68 to 13.75) | 12.44 (11.84 to 13.03) | 9.12 (8.54 to 9.69) | 6.42 (5.96 to 6.87) | 7.55 (7.01 to 8.09) | 8.63 (8.08 to 9.18) |
|  |  | Mid-level of household income | 14.83 (14.39 to 15.26) | 14.22 (13.74 to 14.71) | 12.02 (11.59 to 12.45) | 13.29 (12.84 to 13.73) | 10.64 (10.23 to 11.06) | 10.43 (10.02 to 10.84) | 9.79 (9.39 to 10.20) | 9.25 (8.86 to 9.65) | 8.10 (7.72 to 8.49) | 5.84 (5.50 to 6.19) | 4.37 (4.06 to 4.67) | 4.76 (4.44 to 5.08) | 5.04 (4.72 to 5.36) |
|  |  | High-level of household income | 12.29 (11.08 to 13.50) | 12.63 (11.38 to 13.89) | 9.58 (8.57 to 10.59) | 11.26 (10.20 to 12.32) | 8.07 (6.95 to 9.18) | 8.84 (7.50 to 10.18) | 8.86 (7.76 to 9.97) | 8.15 (6.97 to 9.33) | 7.45 (6.46 to 8.44) | 5.16 (4.37 to 5.95) | 3.84 (3.13 to 4.54) | 4.54 (3.61 to 5.48) | 3.23 (2.61 to 3.85) |
|  | College or higher education | Low-level of household income | 21.45 (20.37 to 22.53) | 16.56 (15.46 to 17.67) | 14.79 (13.71 to 15.86) | 16.41 (15.29 to 17.54) | 13.91 (13.07 to 14.74) | 13.77 (12.90 to 14.64) | 13.05 (12.24 to 13.86) | 12.68 (11.82 to 13.55) | 11.73 (10.60 to 12.87) | 8.43 (7.42 to 9.44) | 6.70 (5.86 to 7.53) | 8.12 (7.24 to 8.99) | 9.09 (8.13 to 10.05) |
|  |  | Mid-level of household income | 17.67 (17.35 to 17.98) | 13.59 (13.31 to 13.87) | 12.05 (11.79 to 12.32) | 13.61 (13.33 to 13.89) | 11.78 (11.53 to 12.03) | 11.99 (11.74 to 12.24) | 11.81 (11.55 to 12.06) | 10.86 (10.61 to 11.11) | 9.10 (8.82 to 9.37) | 6.14 (5.89 to 6.38) | 5.65 (5.42 to 5.89) | 5.30 (5.07 to 5.52) | 5.40 (5.17 to 5.63) |
|  |  | High-level of household income | 14.99 (14.44 to 15.53) | 11.36 (10.92 to 11.80) | 10.29 (9.91 to 10.67) | 11.89 (11.49 to 12.29) | 10.38 (9.97 to 10.79) | 10.47 (10.06 to 10.87) | 10.64 (10.25 to 11.03) | 9.76 (9.40 to 10.11) | 7.86 (7.55 to 8.17) | 5.26 (4.99 to 5.53) | 4.67 (4.40 to 4.94) | 4.38 (4.13 to 4.63) | 4.73 (4.49 to 4.98) |
| Smoking status | Smoker | Low-level of household income | 22.60 (21.43 to 23.77) | 19.55 (18.28 to 20.82) | 16.81 (15.50 to 18.11) | 20.56 (19.10 to 22.01) | 16.54 (15.41 to 17.67) | 15.43 (14.18 to 16.69) | 16.09 (14.81 to 17.36) | 16.47 (15.10 to 17.83) | 14.19 (12.64 to 15.74) | 11.54 (9.96 to 13.12) | 8.12 (6.83 to 9.41) | 10.85 (9.24 to 12.47) | 12.65 (11.12 to 14.17) |
|  |  | Mid-level of household income | 17.85 (17.35 to 18.34) | 14.57 (14.08 to 15.06) | 12.52 (12.06 to 12.98) | 14.40 (13.89 to 14.90) | 12.33 (11.88 to 12.78) | 12.94 (12.45 to 13.42) | 12.69 (12.21 to 13.17) | 12.16 (11.68 to 12.64) | 10.15 (9.62 to 10.68) | 7.06 (6.58 to 7.54) | 6.90 (6.41 to 7.40) | 6.12 (5.65 to 6.59) | 6.64 (6.14 to 7.14) |
|  |  | High-level of household income | 15.99 (15.01 to 16.98) | 12.42 (11.54 to 13.30) | 10.35 (9.58 to 11.13) | 12.38 (11.57 to 13.19) | 10.85 (9.99 to 11.71) | 11.12 (10.22 to 12.02) | 11.65 (10.79 to 12.50) | 10.34 (9.55 to 11.14) | 9.23 (8.52 to 9.94) | 6.26 (5.62 to 6.90) | 4.90 (4.30 to 5.49) | 5.72 (5.05 to 6.39) | 6.19 (5.57 to 6.81) |
|  | Ex-smoker | Low-level of household income | 18.38 (17.20 to 19.55) | 17.11 (15.88 to 18.33) | 13.60 (12.46 to 14.75) | 13.72 (12.67 to 14.76) | 11.06 (10.15 to 11.98) | 10.72 (9.87 to 11.57) | 10.53 (9.66 to 11.39) | 10.49 (9.61 to 11.38) | 9.31 (8.28 to 10.33) | 7.44 (6.47 to 8.42) | 5.23 (4.35 to 6.10) | 6.88 (5.78 to 7.99) | 7.82 (6.80 to 8.84) |
|  |  | Mid-level of household income | 14.13 (13.54 to 14.72) | 11.89 (11.33 to 12.46) | 9.93 (9.40 to 10.46) | 11.45 (10.91 to 11.99) | 8.88 (8.42 to 9.35) | 9.59 (9.13 to 10.04) | 8.98 (8.52 to 9.43) | 8.73 (8.29 to 9.18) | 7.01 (6.56 to 7.45) | 5.32 (4.91 to 5.72) | 4.69 (4.29 to 5.09) | 4.42 (4.02 to 4.81) | 4.28 (3.95 to 4.61) |
|  |  | High-level of household income | 12.11 (11.06 to 13.15) | 10.54 (9.57 to 11.51) | 8.56 (7.76 to 9.37) | 9.57 (8.77 to 10.38) | 8.72 (7.80 to 9.64) | 7.70 (6.94 to 8.45) | 8.62 (7.82 to 9.41) | 7.98 (7.25 to 8.71) | 6.69 (6.03 to 7.34) | 4.45 (3.92 to 4.97) | 3.89 (3.37 to 4.40) | 3.39 (2.91 to 3.87) | 4.31 (3.87 to 4.76) |
|  | Non-smoker | Low-level of household income | 21.53 (20.90 to 22.15) | 20.38 (19.70 to 21.05) | 18.03 (17.36 to 18.70) | 19.12 (18.45 to 19.78) | 15.43 (14.87 to 15.99) | 15.45 (14.85 to 16.04) | 14.44 (13.89 to 15.00) | 12.99 (12.45 to 13.54) | 12.61 (11.94 to 13.27) | 8.76 (8.17 to 9.35) | 6.52 (6.04 to 7.00) | 7.29 (6.78 to 7.80) | 8.14 (7.57 to 8.70) |
|  |  | Mid-level of household income | 17.39 (17.07 to 17.72) | 13.84 (13.53 to 14.15) | 12.40 (12.11 to 12.70) | 13.74 (13.43 to 14.04) | 11.97 (11.70 to 12.24) | 11.85 (11.58 to 12.13) | 11.68 (11.40 to 11.95) | 10.51 (10.24 to 10.77) | 8.95 (8.66 to 9.23) | 6.00 (5.74 to 6.25) | 5.07 (4.84 to 5.30) | 5.12 (4.89 to 5.36) | 5.28 (5.05 to 5.52) |
|  |  | High-level of household income | 14.91 (14.30 to 15.51) | 11.37 (10.87 to 11.87) | 10.61 (10.16 to 11.07) | 12.22 (11.75 to 12.68) | 10.37 (9.89 to 10.85) | 10.83 (10.35 to 11.31) | 10.70 (10.25 to 11.16) | 9.91 (9.48 to 10.33) | 7.70 (7.34 to 8.05) | 5.21 (4.90 to 5.52) | 4.74 (4.43 to 5.05) | 4.34 (4.06 to 4.62) | 4.34 (4.07 to 4.61) |
| Alcohol consumption | Non-drinker and 1day/month | Low-level of household income | 21.55 (20.95 to 22.15) | 20.13 (19.48 to 20.78) | 17.94 (17.30 to 18.58) | 19.26 (18.61 to 19.90) | 15.05 (14.52 to 15.58) | 14.72 (14.18 to 15.26) | 13.90 (13.37 to 14.42) | 12.98 (12.46 to 13.51) | 12.24 (11.63 to 12.85) | 8.88 (8.33 to 9.43) | 6.44 (5.99 to 6.89) | 7.50 (7.00 to 8.00) | 8.54 (8.01 to 9.07) |
|  |  | Mid-level of household income | 15.94 (15.61 to 16.27) | 13.39 (13.06 to 13.72) | 11.73 (11.43 to 12.03) | 13.31 (12.99 to 13.64) | 11.37 (11.07 to 11.67) | 11.31 (11.01 to 11.60) | 10.90 (10.61 to 11.19) | 9.98 (9.69 to 10.27) | 8.17 (7.88 to 8.46) | 5.81 (5.56 to 6.07) | 5.01 (4.77 to 5.24) | 5.04 (4.81 to 5.27) | 5.13 (4.91 to 5.36) |
|  |  | High-level of household income | 14.10 (13.43 to 14.77) | 10.67 (10.11 to 11.23) | 9.95 (9.45 to 10.45) | 11.35 (10.83 to 11.86) | 9.83 (9.27 to 10.39) | 10.00 (9.45 to 10.55) | 9.87 (9.34 to 10.41) | 9.05 (8.56 to 9.53) | 7.33 (6.91 to 7.75) | 5.01 (4.65 to 5.36) | 4.54 (4.20 to 4.89) | 3.99 (3.69 to 4.28) | 4.35 (4.05 to 4.65) |
|  | 2–9 days/month | Low-level of household income | 20.86 (19.68 to 22.03) | 18.48 (17.26 to 19.70) | 15.09 (13.92 to 16.27) | 16.55 (15.28 to 17.83) | 14.76 (13.74 to 15.78) | 13.86 (12.81 to 14.90) | 13.85 (12.82 to 14.88) | 13.25 (12.17 to 14.34) | 12.16 (10.90 to 13.41) | 8.87 (7.54 to 10.19) | 6.97 (5.77 to 8.17) | 8.10 (6.72 to 9.48) | 9.40 (8.01 to 10.79) |
|  |  | Mid-level of household income | 18.24 (17.83 to 18.66) | 13.88 (13.50 to 14.25) | 12.23 (11.86 to 12.60) | 13.56 (13.17 to 13.94) | 11.69 (11.36 to 12.02) | 11.92 (11.58 to 12.26) | 11.80 (11.46 to 12.14) | 11.05 (10.71 to 11.38) | 9.55 (9.17 to 9.92) | 6.11 (5.78 to 6.45) | 5.71 (5.36 to 6.06) | 5.05 (4.73 to 5.38) | 5.46 (5.11 to 5.80) |
|  |  | High-level of household income | 15.21 (14.46 to 15.96) | 12.10 (11.50 to 12.70) | 10.38 (9.86 to 10.90) | 12.20 (11.64 to 12.75) | 10.39 (9.83 to 10.95) | 10.52 (9.99 to 11.06) | 10.90 (10.37 to 11.42) | 10.08 (9.58 to 10.57) | 8.05 (7.63 to 8.47) | 5.33 (4.95 to 5.70) | 4.58 (4.21 to 4.95) | 4.62 (4.24 to 5.01) | 4.84 (4.50 to 5.19) |
|  | Over 10 days/month | Low-level of household income | 19.11 (17.44 to 20.79) | 18.30 (16.71 to 19.89) | 13.67 (12.01 to 15.32) | 15.10 (13.41 to 16.78) | 13.39 (11.97 to 14.81) | 14.55 (12.86 to 16.25) | 14.29 (12.55 to 16.04) | 12.93 (11.15 to 14.71) | 12.13 (10.10 to 14.16) | 9.36 (7.29 to 11.44) | 6.27 (4.58 to 7.96) | 11.09 (8.37 to 13.81) | 11.03 (8.25 to 13.82) |
|  |  | Mid-level of household income | 18.69 (17.78 to 19.61) | 15.30 (14.38 to 16.21) | 13.34 (12.45 to 14.22) | 15.09 (14.18 to 16.00) | 12.19 (11.41 to 12.97) | 13.08 (12.30 to 13.87) | 12.93 (12.12 to 13.74) | 11.72 (10.95 to 12.50) | 10.36 (9.55 to 11.18) | 8.00 (7.13 to 8.87) | 7.03 (6.10 to 7.95) | 7.55 (6.55 to 8.56) | 6.46 (5.63 to 7.29) |
|  |  | High-level of household income | 16.51 (14.61 to 18.40) | 12.53 (10.90 to 14.16) | 11.36 (9.83 to 12.90) | 12.82 (11.35 to 14.28) | 11.62 (9.99 to 13.25) | 11.61 (10.02 to 13.20) | 12.53 (11.03 to 14.04) | 10.74 (9.40 to 12.08) | 9.65 (8.51 to 10.79) | 6.54 (5.51 to 7.57) | 5.78 (4.68 to 6.89) | 7.20 (5.86 to 8.54) | 5.97 (4.89 to 7.05) |
| Health-level | High | Low-level of household income | 13.33 (12.46 to 14.20) | 9.74 (8.80 to 10.68) | 8.47 (7.59 to 9.35) | 9.19 (8.24 to 10.14) | 7.76 (7.01 to 8.51) | 8.03 (7.21 to 8.85) | 7.06 (6.30 to 7.82) | 6.93 (6.11 to 7.76) | 5.35 (4.53 to 6.18) | 4.02 (3.15 to 4.89) | 4.03 (3.33 to 4.72) | 3.67 (2.91 to 4.44) | 4.65 (3.71 to 5.59) |
|  |  | Mid-level of household income | 12.72 (12.40 to 13.04) | 9.59 (9.29 to 9.89) | 8.31 (8.02 to 8.60) | 9.55 (9.24 to 9.86) | 8.05 (7.76 to 8.33) | 8.39 (8.11 to 8.67) | 8.10 (7.81 to 8.39) | 7.51 (7.22 to 7.80) | 5.60 (5.32 to 5.89) | 4.30 (4.00 to 4.60) | 3.79 (3.56 to 4.01) | 3.38 (3.15 to 3.62) | 3.60 (3.35 to 3.84) |
|  |  | High-level of household income | 11.24 (10.66 to 11.81) | 7.83 (7.36 to 8.30) | 7.31 (6.89 to 7.73) | 8.73 (8.28 to 9.19) | 7.35 (6.89 to 7.81) | 7.96 (7.48 to 8.43) | 7.38 (6.94 to 7.82) | 6.99 (6.58 to 7.40) | 5.23 (4.87 to 5.59) | 3.59 (3.28 to 3.89) | 3.29 (3.02 to 3.56) | 3.23 (2.95 to 3.51) | 3.43 (3.17 to 3.70) |
|  | Middle | Low-level of household income | 18.71 (17.82 to 19.60) | 16.92 (15.97 to 17.88) | 12.83 (12.00 to 13.66) | 14.33 (13.47 to 15.19) | 11.67 (10.98 to 12.37) | 12.09 (11.34 to 12.85) | 11.05 (10.36 to 11.74) | 10.36 (9.66 to 11.06) | 9.42 (8.62 to 10.21) | 6.37 (5.62 to 7.12) | 5.11 (4.53 to 5.68) | 6.12 (5.41 to 6.83) | 6.40 (5.66 to 7.14) |
|  |  | Mid-level of household income | 20.35 (19.92 to 20.77) | 15.98 (15.58 to 16.38) | 13.63 (13.27 to 13.99) | 15.34 (14.96 to 15.73) | 13.18 (12.85 to 13.51) | 13.34 (13.00 to 13.67) | 13.16 (12.83 to 13.50) | 12.06 (11.73 to 12.38) | 9.79 (9.43 to 10.14) | 6.21 (5.93 to 6.50) | 6.17 (5.85 to 6.49) | 5.52 (5.22 to 5.81) | 5.78 (5.48 to 6.08) |
|  |  | High-level of household income | 18.86 (17.99 to 19.74) | 14.92 (14.21 to 15.63) | 12.78 (12.17 to 13.38) | 14.20 (13.60 to 14.80) | 12.48 (11.83 to 13.13) | 12.14 (11.52 to 12.75) | 13.04 (12.42 to 13.65) | 11.83 (11.27 to 12.39) | 9.31 (8.87 to 9.75) | 6.12 (5.71 to 6.52) | 6.17 (5.70 to 6.63) | 5.19 (4.79 to 5.58) | 5.57 (5.21 to 5.93) |
|  | Low | Low-level of household income | 27.79 (26.97 to 28.60) | 27.17 (26.30 to 28.04) | 24.56 (23.63 to 25.49) | 25.71 (24.82 to 26.59) | 21.13 (20.36 to 21.90) | 19.87 (19.10 to 20.65) | 19.64 (18.86 to 20.43) | 17.86 (17.11 to 18.60) | 17.84 (16.89 to 18.79) | 12.38 (11.58 to 13.19) | 10.36 (9.51 to 11.20) | 11.67 (10.82 to 12.52) | 12.37 (11.54 to 13.20) |
|  |  | Mid-level of household income | 22.88 (22.18 to 23.58) | 21.00 (20.26 to 21.74) | 19.29 (18.59 to 19.99) | 20.59 (19.88 to 21.31) | 17.72 (17.03 to 18.41) | 17.58 (16.87 to 18.29) | 16.45 (15.78 to 17.11) | 15.11 (14.45 to 15.77) | 13.95 (13.31 to 14.59) | 9.22 (8.69 to 9.76) | 9.39 (8.68 to 10.10) | 9.24 (8.60 to 9.87) | 8.34 (7.81 to 8.88) |
|  |  | High-level of household income | 20.13 (18.44 to 21.82) | 19.09 (17.37 to 20.81) | 16.40 (14.94 to 17.86) | 20.46 (18.86 to 22.06) | 16.61 (15.02 to 18.19) | 16.99 (15.35 to 18.63) | 17.98 (16.43 to 19.53) | 15.69 (14.23 to 17.16) | 14.39 (13.13 to 15.66) | 9.42 (8.30 to 10.53) | 9.52 (7.99 to 11.06) | 8.14 (7.05 to 9.22) | 8.68 (7.54 to 9.82) |

**Abbreviations:** BMI, body mass index; CI, confidence interval; KCHS, Korea Community Health Survey.

* According to the Asian–Pacific guidelines, the BMI is divided into four groups: underweight (<18.5 kg/m^2^), normal (18.5–22.9 kg/m^2^), overweight (23.0–24.9 kg/m^2^), and obese (≥25.0 kg/m^2^).

**Table S2**. National trends in the prevalence of unmet healthcare needs and odds ratios β-coefficients before and during the COVID-19 pandemic, presented as weighted percentages with 95% confidence intervals, using data obtained from KCHS.

| Variables | | | Pre-pandemic | | | | | Pandemic | | | Trend in the pre-pandemic era, β (95% CI) | Trend in the pandemic era, β (95% CI) | Trend difference, β_diff_ (95% CI) |
| --- | --- | --- | --- | --- | --- | --- | --- | --- | --- | --- | --- | --- | --- |
|  |  |  | 2010-2011 | 2012-2013 | 2014-2015 | 2016-2017 | 2018-2019 | 2020 | 2021 | 2022 |  |  |  |
| Overall | | Low-level of household income | 20.43 (19.95 to 20.91) | 17.70 (17.26 to 18.13) | 14.69 (14.32 to 15.06) | 13.48 (13.12 to 13.84) | 10.65 (10.23 to 11.07) | 6.51 (6.09 to 6.94) | 7.75 (7.28 to 8.23) | 8.79 (8.29 to 9.29) | **-2.37 (-2.51 to -2.23)** | **-0.71 (-0.92 to -0.49)** | **1.66 (1.41 to 1.92)** |
|  |  | Mid-level of household income | 15.37 (15.15 to 15.60) | 12.80 (12.61 to 12.99) | 11.64 (11.46 to 11.81) | 11.00 (10.83 to 11.17) | 7.54 (7.36 to 7.72) | 5.34 (5.15 to 5.54) | 5.17 (4.98 to 5.36) | 5.31 (5.12 to 5.50) | **-1.70 (-1.77 to -1.64)** | **-0.82 (-0.91 to -0.73)** | **0.88 (0.77 to 0.99)** |
|  |  | High-level of household income | 12.97 (12.59 to 13.34) | 11.08 (10.79 to 11.37) | 10.29 (9.98 to 10.59) | 10.07 (9.79 to 10.34) | 6.58 (6.36 to 6.79) | 4.62 (4.36 to 4.87) | 4.39 (4.15 to 4.63) | 4.64 (4.41 to 4.88) | **-1.41 (-1.50 to -1.32)** | **-0.70 (-0.81 to -0.60)** | **0.71 (0.57 to 0.85)** |
| Residential area | Urban | Low-level of household income | 21.73 (21.04 to 22.42) | 17.76 (17.13 to 18.39) | 14.61 (14.10 to 15.12) | 13.46 (12.96 to 13.95) | 10.69 (10.10 to 11.28) | 6.13 (5.56 to 6.70) | 7.72 (7.10 to 8.34) | 8.91 (8.24 to 9.57) | **-2.65 (-2.85 to -2.45)** | **-0.66 (-0.95 to -0.36)** | **1.99 (1.64 to 2.35)** |
|  |  | Mid-level of household income | 15.70 (15.44 to 15.97) | 12.98 (12.76 to 13.20) | 11.67 (11.47 to 11.87) | 11.07 (10.87 to 11.27) | 7.54 (7.33 to 7.74) | 5.31 (5.09 to 5.54) | 5.13 (4.91 to 5.35) | 5.22 (5.01 to 5.44) | **-1.78 (-1.86 to -1.71)** | **-0.84 (-0.95 to -0.74)** | **0.94 (0.81 to 1.07)** |
|  |  | High-level of household income | 12.92 (12.51 to 13.33) | 11.18 (10.87 to 11.50) | 10.12 (9.80 to 10.44) | 10.00 (9.71 to 10.30) | 6.56 (6.33 to 6.80) | 4.47 (4.19 to 4.74) | 4.27 (4.01 to 4.53) | 4.53 (4.28 to 4.78) | **-1.42 (-1.52 to -1.32)** | **-0.74 (-0.86 to -0.63)** | **0.68 (0.53 to 0.83)** |
|  | Rural | Low-level of household income | 18.29 (17.74 to 18.84) | 17.59 (17.09 to 18.09) | 14.86 (14.39 to 15.32) | 13.53 (13.08 to 13.98) | 10.57 (10.09 to 11.06) | 7.29 (6.72 to 7.86) | 7.84 (7.21 to 8.48) | 8.52 (7.88 to 9.17) | **-1.91 (-2.08 to -1.74)** | **-0.80 (-1.06 to -0.54)** | **1.10 (0.79 to 1.42)** |
|  |  | Mid-level of household income | 13.95 (13.54 to 14.36) | 12.01 (11.67 to 12.35) | 11.48 (11.14 to 11.82) | 10.70 (10.38 to 11.02) | 7.56 (7.23 to 7.89) | 5.47 (5.11 to 5.83) | 5.33 (4.96 to 5.69) | 5.70 (5.33 to 6.06) | **-1.36 (-1.48 to -1.24)** | **-0.71 (-0.88 to -0.55)** | **0.65 (0.44 to 0.85)** |
|  |  | High-level of household income | 13.32 (12.48 to 14.16) | 10.30 (9.67 to 10.93) | 11.65 (10.67 to 12.64) | 10.55 (9.91 to 11.19) | 6.66 (6.11 to 7.21) | 5.69 (4.97 to 6.41) | 5.24 (4.61 to 5.87) | 5.41 (4.82 to 5.99) | **-1.34 (-1.55 to -1.12)** | **-0.45 (-0.72 to -0.18)** | **0.88 (0.54 to 1.23)** |
| BMI | 1 | Low-level of household income | 23.95 (22.90 to 25.01) | 21.60 (20.67 to 22.52) | 18.58 (17.61 to 19.54) | 16.46 (15.55 to 17.36) | 13.89 (12.89 to 14.89) | 7.81 (6.04 to 9.59) | 9.20 (7.66 to 10.73) | 11.34 (9.71 to 12.97) | **-2.53 (-2.86 to -2.21)** | **-1.25 (-1.86 to -0.63)** | **1.29 (0.59 to 1.98)** |
|  |  | Mid-level of household income | 17.30 (16.53 to 18.07) | 15.02 (14.30 to 15.74) | 13.25 (12.57 to 13.94) | 12.66 (11.93 to 13.38) | 8.17 (7.55 to 8.79) | 7.39 (6.28 to 8.49) | 6.47 (5.49 to 7.45) | 5.82 (4.95 to 6.70) | **-2.04 (-2.27 to -1.81)** | **-0.80 (-1.16 to -0.44)** | **1.24 (0.82 to 1.66)** |
|  |  | High-level of household income | 14.08 (12.72 to 15.43) | 13.11 (11.94 to 14.28) | 11.48 (10.22 to 12.73) | 11.95 (10.72 to 13.17) | 6.54 (5.75 to 7.34) | 5.12 (3.85 to 6.39) | 5.02 (3.88 to 6.16) | 5.26 (4.16 to 6.35) | **-1.76 (-2.10 to -1.42)** | **-0.49 (-0.94 to -0.04)** | **1.27 (0.70 to 1.83)** |
|  | 2 | Low-level of household income | 20.15 (19.46 to 20.84) | 16.80 (16.15 to 17.44) | 14.46 (13.91 to 15.01) | 13.37 (12.83 to 13.92) | 10.78 (10.06 to 11.50) | 6.70 (6.04 to 7.35) | 7.48 (6.77 to 8.18) | 8.51 (7.74 to 9.28) | **-2.23 (-2.45 to -2.01)** | **-0.80 (-1.15 to -0.46)** | **1.42 (1.01 to 1.83)** |
|  |  | Mid-level of household income | 15.58 (15.27 to 15.89) | 12.91 (12.64 to 13.18) | 11.91 (11.66 to 12.16) | 11.18 (10.93 to 11.44) | 7.72 (7.44 to 8.00) | 5.21 (4.91 to 5.51) | 5.28 (4.98 to 5.59) | 5.36 (5.06 to 5.65) | **-1.68 (-1.78 to -1.59)** | **-0.85 (-0.98 to -0.71)** | **0.84 (0.67 to 1.00)** |
|  |  | High-level of household income | 13.17 (12.67 to 13.68) | 11.21 (10.82 to 11.61) | 10.71 (10.27 to 11.14) | 10.13 (9.74 to 10.52) | 6.60 (6.29 to 6.92) | 4.72 (4.34 to 5.10) | 4.17 (3.80 to 4.53) | 4.69 (4.35 to 5.04) | **-1.44 (-1.57 to -1.31)** | **-0.72 (-0.88 to -0.57)** | **0.71 (0.51 to 0.92)** |
|  | 3 | Low-level of household income | 18.44 (17.52 to 19.36) | 15.71 (14.85 to 16.57) | 13.33 (12.62 to 14.05) | 12.12 (11.43 to 12.82) | 9.27 (8.45 to 10.08) | 5.85 (5.03 to 6.66) | 6.53 (5.61 to 7.44) | 7.76 (6.85 to 8.67) | **-2.17 (-2.45 to -1.90)** | **-0.59 (-0.99 to -0.18)** | **1.59 (1.10 to 2.07)** |
|  |  | Mid-level of household income | 14.35 (13.96 to 14.74) | 12.06 (11.72 to 12.41) | 10.68 (10.36 to 10.99) | 10.22 (9.90 to 10.54) | 7.12 (6.80 to 7.45) | 4.96 (4.60 to 5.32) | 4.73 (4.38 to 5.08) | 5.12 (4.76 to 5.49) | **-1.59 (-1.71 to -1.48)** | **-0.76 (-0.92 to -0.60)** | **0.83 (0.64 to 1.03)** |
|  |  | High-level of household income | 12.24 (11.56 to 12.91) | 10.32 (9.79 to 10.86) | 9.79 (9.23 to 10.36) | 9.53 (9.02 to 10.05) | 6.10 (5.68 to 6.52) | 4.34 (3.87 to 4.81) | 4.70 (4.20 to 5.21) | 4.75 (4.27 to 5.22) | **-1.34 (-1.51 to -1.17)** | **-0.46 (-0.67 to -0.26)** | **0.87 (0.61 to 1.14)** |
|  | 4 | Low-level of household income | 20.55 (19.61 to 21.49) | 18.29 (17.35 to 19.24) | 14.11 (13.40 to 14.83) | 13.40 (12.69 to 14.11) | 9.94 (9.23 to 10.64) | 6.55 (5.83 to 7.27) | 8.80 (7.82 to 9.78) | 9.26 (8.29 to 10.23) | **-2.59 (-2.85 to -2.32)** | -0.24 (-0.63 to 0.16) | **2.35 (1.87 to 2.82)** |
|  |  | Mid-level of household income | 15.48 (15.08 to 15.87) | 12.73 (12.38 to 13.08) | 11.69 (11.38 to 12.01) | 11.08 (10.77 to 11.38) | 7.50 (7.22 to 7.78) | 5.55 (5.22 to 5.89) | 5.18 (4.86 to 5.51) | 5.32 (5.00 to 5.65) | **-1.74 (-1.85 to -1.63)** | **-0.81 (-0.95 to -0.66)** | **0.93 (0.75 to 1.11)** |
|  |  | High-level of household income | 12.96 (12.25 to 13.67) | 11.04 (10.50 to 11.58) | 9.67 (9.10 to 10.23) | 10.05 (9.55 to 10.55) | 6.90 (6.53 to 7.28) | 4.62 (4.17 to 5.06) | 4.36 (3.94 to 4.78) | 4.40 (4.04 to 4.77) | **-1.34 (-1.50 to -1.17)** | **-0.88 (-1.06 to -0.71)** | **0.45 (0.21 to 0.69)** |
| Education | High school or lower education | Low-level of household income | 21.10 (20.57 to 21.63) | 18.64 (18.15 to 19.14) | 15.17 (14.74 to 15.60) | 13.81 (13.39 to 14.22) | 10.86 (10.40 to 11.31) | 6.42 (5.96 to 6.87) | 7.55 (7.01 to 8.09) | 8.63 (8.08 to 9.18) | **-2.53 (-2.68 to -2.37)** | **-0.86 (-1.10 to -0.63)** | **1.66 (1.38 to 1.95)** |
|  |  | Mid-level of household income | 14.52 (14.13 to 14.91) | 12.65 (12.31 to 13.00) | 10.54 (10.22 to 10.85) | 9.53 (9.22 to 9.84) | 6.98 (6.70 to 7.27) | 4.37 (4.06 to 4.67) | 4.76 (4.44 to 5.08) | 5.04 (4.72 to 5.36) | **-1.82 (-1.93 to -1.71)** | **-0.71 (-0.85 to -0.57)** | **1.11 (0.93 to 1.29)** |
|  |  | High-level of household income | 12.47 (11.46 to 13.47) | 10.47 (9.67 to 11.26) | 8.45 (7.53 to 9.36) | 8.50 (7.64 to 9.36) | 6.31 (5.64 to 6.97) | 3.84 (3.13 to 4.54) | 4.54 (3.61 to 5.48) | 3.23 (2.61 to 3.85) | **-1.43 (-1.69 to -1.16)** | **-0.97 (-1.28 to -0.65)** | **0.46 (0.05 to 0.87)** |
|  | College or higher education | Low-level of household income | 19.05 (18.17 to 19.93) | 15.61 (14.78 to 16.44) | 13.84 (13.19 to 14.50) | 12.87 (12.23 to 13.51) | 10.19 (9.35 to 11.02) | 6.70 (5.86 to 7.53) | 8.12 (7.24 to 8.99) | 9.09 (8.13 to 10.05) | **-2.04 (-2.30 to -1.77)** | -0.39 (-0.81 to 0.02) | **1.64 (1.15 to 2.14)** |
|  |  | Mid-level of household income | 15.61 (15.35 to 15.86) | 12.84 (12.62 to 13.06) | 11.89 (11.69 to 12.08) | 11.34 (11.14 to 11.54) | 7.72 (7.51 to 7.92) | 5.65 (5.42 to 5.89) | 5.30 (5.07 to 5.52) | 5.40 (5.17 to 5.63) | **-1.67 (-1.75 to -1.60)** | **-0.85 (-0.96 to -0.75)** | **0.82 (0.69 to 0.95)** |
|  |  | High-level of household income | 13.01 (12.62 to 13.40) | 11.13 (10.83 to 11.44) | 10.42 (10.11 to 10.74) | 10.17 (9.88 to 10.45) | 6.59 (6.37 to 6.82) | 4.67 (4.40 to 4.94) | 4.38 (4.13 to 4.63) | 4.73 (4.49 to 4.98) | **-1.42 (-1.51 to -1.32)** | **-0.69 (-0.80 to -0.58)** | **0.73 (0.58 to 0.88)** |
| Smoking status | Smoker | Low-level of household income | 21.13 (20.11 to 22.16) | 18.64 (17.60 to 19.68) | 16.03 (15.12 to 16.94) | 16.27 (15.27 to 17.27) | 12.97 (11.76 to 14.18) | 8.12 (6.83 to 9.41) | 10.85 (9.24 to 12.47) | 12.65 (11.12 to 14.17) | **-1.85 (-2.20 to -1.50)** | -0.17 (-0.80 to 0.47) | **1.69 (0.96 to 2.41)** |
|  |  | Mid-level of household income | 16.20 (15.80 to 16.61) | 13.45 (13.08 to 13.83) | 12.62 (12.26 to 12.98) | 12.43 (12.06 to 12.80) | 8.76 (8.36 to 9.17) | 6.90 (6.41 to 7.40) | 6.12 (5.65 to 6.59) | 6.64 (6.14 to 7.14) | **-1.53 (-1.66 to -1.40)** | **-0.84 (-1.06 to -0.63)** | **0.69 (0.44 to 0.94)** |
|  |  | High-level of household income | 14.08 (13.32 to 14.85) | 11.41 (10.81 to 12.02) | 10.98 (10.32 to 11.65) | 10.96 (10.33 to 11.58) | 7.85 (7.33 to 8.37) | 4.90 (4.30 to 5.49) | 5.72 (5.05 to 6.39) | 6.19 (5.57 to 6.81) | **-1.29 (-1.49 to -1.09)** | **-0.62 (-0.88 to -0.35)** | **0.67 (0.34 to 1.00)** |
|  | Ex-smoker | Low-level of household income | 17.71 (16.75 to 18.68) | 13.66 (12.81 to 14.51) | 10.89 (10.19 to 11.59) | 10.51 (9.84 to 11.18) | 8.41 (7.66 to 9.16) | 5.23 (4.35 to 6.10) | 6.88 (5.78 to 7.99) | 7.82 (6.80 to 8.84) | **-2.18 (-2.45 to -1.91)** | -0.22 (-0.63 to 0.19) | **1.96 (1.47 to 2.45)** |
|  |  | Mid-level of household income | 12.95 (12.48 to 13.41) | 10.70 (10.28 to 11.12) | 9.25 (8.90 to 9.61) | 8.86 (8.51 to 9.20) | 6.14 (5.81 to 6.46) | 4.69 (4.29 to 5.09) | 4.42 (4.02 to 4.81) | 4.28 (3.95 to 4.61) | **-1.52 (-1.65 to -1.39)** | **-0.65 (-0.80 to -0.49)** | **0.87 (0.67 to 1.07)** |
|  |  | High-level of household income | 11.22 (10.42 to 12.03) | 9.09 (8.49 to 9.69) | 8.17 (7.54 to 8.80) | 8.28 (7.71 to 8.84) | 5.50 (5.06 to 5.93) | 3.89 (3.37 to 4.40) | 3.39 (2.91 to 3.87) | 4.31 (3.87 to 4.76) | **-1.23 (-1.42 to -1.04)** | **-0.47 (-0.68 to -0.27)** | **0.76 (0.48 to 1.04)** |
|  | Non-smoker | Low-level of household income | 20.95 (20.40 to 21.51) | 18.58 (18.06 to 19.09) | 15.44 (14.99 to 15.89) | 13.72 (13.30 to 14.15) | 10.79 (10.30 to 11.28) | 6.52 (6.04 to 7.00) | 7.29 (6.78 to 7.80) | 8.14 (7.57 to 8.70) | **-2.51 (-2.67 to -2.34)** | **-1.00 (-1.25 to -0.75)** | **1.51 (1.21 to 1.81)** |
|  |  | Mid-level of household income | 15.60 (15.33 to 15.88) | 13.08 (12.84 to 13.31) | 11.91 (11.70 to 12.13) | 11.09 (10.88 to 11.31) | 7.56 (7.35 to 7.78) | 5.07 (4.84 to 5.30) | 5.12 (4.89 to 5.36) | 5.28 (5.05 to 5.52) | **-1.76 (-1.84 to -1.68)** | **-0.84 (-0.95 to -0.73)** | **0.92 (0.79 to 1.06)** |
|  |  | High-level of household income | 13.00 (12.56 to 13.44) | 11.46 (11.11 to 11.81) | 10.61 (10.24 to 10.97) | 10.28 (9.94 to 10.61) | 6.50 (6.24 to 6.76) | 4.74 (4.43 to 5.05) | 4.34 (4.06 to 4.62) | 4.34 (4.07 to 4.61) | **-1.47 (-1.58 to -1.36)** | **-0.77 (-0.89 to -0.64)** | **0.70 (0.54 to 0.87)** |
| Alcohol consumption | Non-drinker and 1day/month | Low-level of household income | 20.84 (20.31 to 21.38) | 18.60 (18.11 to 19.10) | 14.89 (14.47 to 15.31) | 13.44 (13.04 to 13.85) | 10.62 (10.17 to 11.07) | 6.44 (5.99 to 6.89) | 7.50 (7.00 to 8.00) | 8.54 (8.01 to 9.07) | **-2.56 (-2.71 to -2.40)** | **-0.78 (-1.01 to -0.55)** | **1.78 (1.50 to 2.06)** |
|  |  | Mid-level of household income | 14.67 (14.39 to 14.95) | 12.52 (12.28 to 12.77) | 11.34 (11.11 to 11.57) | 10.45 (10.22 to 10.67) | 7.02 (6.80 to 7.23) | 5.01 (4.77 to 5.24) | 5.04 (4.81 to 5.27) | 5.13 (4.91 to 5.36) | **-1.71 (-1.79 to -1.63)** | **-0.67 (-0.78 to -0.57)** | **1.03 (0.90 to 1.17)** |
|  |  | High-level of household income | 12.30 (11.81 to 12.79) | 10.68 (10.29 to 11.06) | 9.92 (9.50 to 10.34) | 9.44 (9.05 to 9.82) | 6.17 (5.88 to 6.46) | 4.54 (4.20 to 4.89) | 3.99 (3.69 to 4.28) | 4.35 (4.05 to 4.65) | **-1.39 (-1.51 to -1.27)** | **-0.67 (-0.81 to -0.53)** | **0.72 (0.53 to 0.90)** |
|  | 2–9 days/month | Low-level of household income | 19.67 (18.67 to 20.67) | 15.83 (14.88 to 16.78) | 14.33 (13.53 to 15.13) | 13.56 (12.73 to 14.39) | 10.67 (9.65 to 11.69) | 6.97 (5.77 to 8.17) | 8.10 (6.72 to 9.48) | 9.40 (8.01 to 10.79) | **-2.02 (-2.33 to -1.70)** | -0.56 (-1.12 to 0.00) | **1.46 (0.81 to 2.10)** |
|  |  | Mid-level of household income | 15.99 (15.66 to 16.32) | 12.90 (12.61 to 13.19) | 11.81 (11.55 to 12.06) | 11.43 (11.17 to 11.69) | 7.97 (7.69 to 8.26) | 5.71 (5.36 to 6.06) | 5.05 (4.73 to 5.38) | 5.46 (5.11 to 5.80) | **-1.70 (-1.80 to -1.60)** | **-0.97 (-1.12 to -0.82)** | **0.72 (0.55 to 0.90)** |
|  |  | High-level of household income | 13.47 (12.94 to 14.01) | 11.35 (10.94 to 11.76) | 10.46 (10.04 to 10.89) | 10.46 (10.07 to 10.85) | 6.75 (6.44 to 7.06) | 4.58 (4.21 to 4.95) | 4.62 (4.24 to 5.01) | 4.84 (4.50 to 5.19) | **-1.46 (-1.59 to -1.33)** | **-0.69 (-0.85 to -0.54)** | **0.76 (0.56 to 0.97)** |
|  | Over 10 days/month | Low-level of household income | 18.69 (17.29 to 20.09) | 14.37 (13.11 to 15.63) | 13.96 (12.78 to 15.14) | 13.62 (12.30 to 14.95) | 10.94 (9.43 to 12.46) | 6.27 (4.58 to 7.96) | 11.09 (8.37 to 13.81) | 11.03 (8.25 to 13.82) | **-1.60 (-2.06 to -1.15)** | 0.06 (-0.92 to 1.05) | **1.67 (0.58 to 2.75)** |
|  |  | Mid-level of household income | 16.92 (16.18 to 17.66) | 14.22 (13.54 to 14.90) | 12.64 (12.04 to 13.23) | 12.33 (11.73 to 12.94) | 9.35 (8.70 to 10.01) | 7.03 (6.10 to 7.95) | 7.55 (6.55 to 8.56) | 6.46 (5.63 to 7.29) | **-1.67 (-1.89 to -1.45)** | **-0.95 (-1.31 to -0.60)** | **0.72 (0.30 to 1.14)** |
|  |  | High-level of household income | 14.35 (12.94 to 15.76) | 12.15 (11.01 to 13.29) | 11.61 (10.40 to 12.83) | 11.55 (10.48 to 12.61) | 8.19 (7.37 to 9.02) | 5.78 (4.68 to 6.89) | 7.20 (5.86 to 8.54) | 5.97 (4.89 to 7.05) | **-1.33 (-1.67 to -0.98)** | **-0.68 (-1.13 to -0.23)** | **0.65 (0.08 to 1.21)** |
| Health-level | High | Low-level of household income | 11.58 (10.81 to 12.35) | 8.82 (8.12 to 9.52) | 7.89 (7.29 to 8.49) | 7.00 (6.39 to 7.60) | 4.79 (4.13 to 5.44) | 4.03 (3.33 to 4.72) | 3.67 (2.91 to 4.44) | 4.65 (3.71 to 5.59) | **-1.54 (-1.77 to -1.31)** | -0.15 (-0.51 to 0.21) | **1.38 (0.96 to 1.81)** |
|  |  | Mid-level of household income | 11.17 (10.90 to 11.44) | 8.93 (8.69 to 9.17) | 8.22 (8.00 to 8.44) | 7.81 (7.59 to 8.03) | 5.01 (4.77 to 5.25) | 3.79 (3.56 to 4.01) | 3.38 (3.15 to 3.62) | 3.60 (3.35 to 3.84) | **-1.29 (-1.38 to -1.21)** | **-0.53 (-0.64 to -0.41)** | **0.77 (0.63 to 0.91)** |
|  |  | High-level of household income | 9.42 (9.00 to 9.85) | 8.06 (7.72 to 8.40) | 7.67 (7.30 to 8.03) | 7.17 (6.85 to 7.50) | 4.44 (4.18 to 4.70) | 3.29 (3.02 to 3.56) | 3.23 (2.95 to 3.51) | 3.43 (3.17 to 3.70) | **-1.10 (-1.21 to -1.00)** | **-0.35 (-0.48 to -0.23)** | **0.75 (0.59 to 0.91)** |
|  | Middle | Low-level of household income | 17.81 (17.06 to 18.56) | 13.59 (12.94 to 14.25) | 11.87 (11.32 to 12.43) | 10.72 (10.18 to 11.25) | 8.02 (7.41 to 8.62) | 5.11 (4.53 to 5.68) | 6.12 (5.41 to 6.83) | 6.40 (5.66 to 7.14) | **-2.24 (-2.45 to -2.02)** | **-0.56 (-0.88 to -0.25)** | **1.68 (1.29 to 2.06)** |
|  |  | Mid-level of household income | 18.09 (17.75 to 18.43) | 14.50 (14.21 to 14.79) | 13.26 (13.00 to 13.52) | 12.62 (12.36 to 12.88) | 8.07 (7.82 to 8.33) | 6.17 (5.85 to 6.49) | 5.52 (5.22 to 5.81) | 5.78 (5.48 to 6.08) | **-2.14 (-2.23 to -2.04)** | **-0.88 (-1.01 to -0.74)** | **1.26 (1.10 to 1.43)** |
|  |  | High-level of household income | 16.68 (16.06 to 17.30) | 13.53 (13.07 to 13.99) | 12.30 (11.83 to 12.78) | 12.39 (11.95 to 12.83) | 7.74 (7.42 to 8.06) | 6.17 (5.70 to 6.63) | 5.19 (4.79 to 5.58) | 5.57 (5.21 to 5.93) | **-1.93 (-2.08 to -1.79)** | **-0.83 (-0.99 to -0.67)** | **1.10 (0.88 to 1.32)** |
|  | Low | Low-level of household income | 27.48 (26.75 to 28.20) | 25.14 (24.45 to 25.84) | 20.52 (19.91 to 21.12) | 18.75 (18.16 to 19.33) | 15.09 (14.42 to 15.77) | 10.36 (9.51 to 11.20) | 11.67 (10.82 to 12.52) | 12.37 (11.54 to 13.20) | **-3.10 (-3.33 to -2.88)** | **-1.00 (-1.35 to -0.64)** | **2.11 (1.69 to 2.52)** |
|  |  | Mid-level of household income | 21.92 (21.32 to 22.51) | 19.95 (19.40 to 20.50) | 17.65 (17.12 to 18.18) | 15.77 (15.26 to 16.28) | 11.60 (11.15 to 12.06) | 9.39 (8.68 to 10.10) | 9.24 (8.60 to 9.87) | 8.34 (7.81 to 8.88) | **-2.48 (-2.65 to -2.31)** | **-1.09 (-1.33 to -0.86)** | **1.38 (1.09 to 1.67)** |
|  |  | High-level of household income | 19.56 (18.23 to 20.90) | 18.52 (17.36 to 19.68) | 16.80 (15.58 to 18.03) | 16.77 (15.63 to 17.90) | 11.89 (11.01 to 12.78) | 9.52 (7.99 to 11.06) | 8.14 (7.05 to 9.22) | 8.68 (7.54 to 9.82) | **-1.83 (-2.18 to -1.49)** | **-1.24 (-1.70 to -0.78)** | **0.59 (0.01 to 1.17)** |

**Abbreviations**: BMI, body mass index; CI, confidence interval; KCHS, Korea Community Health Survey.

* According to the Asian–Pacific guidelines, the BMI is divided into four groups: underweight (<18.5 kg/m2), normal (18.5–22.9 kg/m2), overweight (23.0–24.9 kg/m2), and obese (≥25.0 kg/m2).

The numbers in bold indicate a significant difference (p<0.05).

**Table S3.** Comparative analysis of Korean’s unmet healthcare needs indicators across various periods by household income levels, KCHS, 2010–2022

| Variables | | | Total | Pre-pandemic | | | | Pandemic | | | |
| --- | --- | --- | --- | --- | --- | --- | --- | --- | --- | --- | --- |
|  |  |  |  | 2010-2011 | 2012-2013 | 2014-2015 | 2016-2017 | 2018-2019 | 2020 | 2021 | 2022 |
| Overall | | Low-level of household income | **1.32 (1.30 to 1.34)** | **1.33 (1.29 to 1.37)** | **1.38 (1.34 to 1.42)** | **1.26 (1.23 to 1.30)** | **1.23 (1.19 to 1.26)** | **1.41 (1.35 to 1.48)** | **1.22 (1.13 to 1.31)** | **1.50 (1.40 to 1.61)** | **1.66 (1.55 to 1.77)** |
|  |  | Mid-level of household income | 1.00 (ref) | 1.00 (ref) | 1.00 (ref) | 1.00 (ref) | 1.00 (ref) | 1.00 (ref) | 1.00 (ref) | 1.00 (ref) | 1.00 (ref) |
|  |  | High-level of household income | **0.79 (0.78 to 0.80)** | **0.84 (0.82 to 0.87)** | **0.87 (0.84 to 0.89)** | **0.88 (0.86 to 0.91)** | **0.92 (0.89 to 0.94)** | **0.87 (0.84 to 0.91)** | **0.87 (0.81 to 0.92)** | **0.85 (0.80 to 0.91)** | **0.87 (0.82 to 0.93)** |
| Residential area | Urban | Low-level of household income | **1.31 (1.29 to 1.34)** | **1.38 (1.34 to 1.44)** | **1.37 (1.32 to 1.42)** | **1.25 (1.20 to 1.30)** | **1.22 (1.17 to 1.27)** | **1.42 (1.33 to 1.51)** | **1.15 (1.04 to 1.28)** | **1.51 (1.37 to 1.65)** | **1.71 (1.57 to 1.86)** |
|  |  | Mid-level of household income | 1.00 (ref) | 1.00 (ref) | 1.00 (ref) | 1.00 (ref) | 1.00 (ref) | 1.00 (ref) | 1.00 (ref) | 1.00 (ref) | 1.00 (ref) |
|  |  | High-level of household income | **0.78 (0.77 to 0.79)** | **0.82 (0.79 to 0.85)** | **0.86 (0.83 to 0.89)** | **0.87 (0.84 to 0.90)** | **0.90 (0.87 to 0.94)** | **0.87 (0.83 to 0.91)** | **0.84 (0.78 to 0.91)** | **0.83 (0.77 to 0.90)** | **0.87 (0.81 to 0.93)** |
|  | Rural | Low-level of household income | **1.36 (1.33 to 1.39)** | **1.31 (1.26 to 1.37)** | **1.47 (1.41 to 1.53)** | **1.29 (1.24 to 1.35)** | **1.26 (1.21 to 1.32)** | **1.40 (1.31 to 1.49)** | **1.33 (1.20 to 1.48)** | **1.47 (1.32 to 1.64)** | **1.50 (1.35 to 1.65)** |
|  |  | Mid-level of household income | 1.00 (ref) | 1.00 (ref) | 1.00 (ref) | 1.00 (ref) | 1.00 (ref) | 1.00 (ref) | 1.00 (ref) | 1.00 (ref) | 1.00 (ref) |
|  |  | High-level of household income | **0.85 (0.83 to 0.88)** | 0.96 (0.89 to 1.02) | **0.86 (0.80 to 0.92)** | 1.02 (0.93 to 1.11) | 0.99 (0.92 to 1.06) | **0.88 (0.80 to 0.97)** | 1.04 (0.90 to 1.20) | 0.98 (0.86 to 1.13) | 0.95 (0.84 to 1.08) |
| BMI^*^ | Underweight | Low-level of household income | **1.45 (1.41 to 1.50)** | **1.38 (1.30 to 1.47)** | **1.44 (1.35 to 1.53)** | **1.40 (1.30 to 1.51)** | **1.30 (1.20 to 1.41)** | **1.70 (1.53 to 1.89)** | 1.06 (0.80 to 1.39) | **1.42 (1.13 to 1.79)** | **1.95 (1.58 to 2.40)** |
|  |  | Mid-level of household income | 1.00 (ref) | 1.00 (ref) | 1.00 (ref) | 1.00 (ref) | 1.00 (ref) | 1.00 (ref) | 1.00 (ref) | 1.00 (ref) | 1.00 (ref) |
|  |  | High-level of household income | **0.77 (0.74 to 0.81)** | **0.81 (0.73 to 0.91)** | **0.87 (0.79 to 0.97)** | **0.87 (0.77 to 0.98)** | 0.94 (0.84 to 1.06) | **0.80 (0.69 to 0.92)** | **0.69 (0.52 to 0.93)** | 0.78 (0.59 to 1.02) | 0.90 (0.70 to 1.17) |
|  | Normal weight | Low-level of household income | **1.26 (1.23 to 1.29)** | **1.29 (1.24 to 1.35)** | **1.30 (1.25 to 1.36)** | **1.21 (1.16 to 1.27)** | **1.20 (1.14 to 1.25)** | **1.40 (1.29 to 1.51)** | **1.29 (1.15 to 1.44)** | **1.42 (1.27 to 1.58)** | **1.59 (1.43 to 1.77)** |
|  |  | Mid-level of household income | 1.00 (ref) | 1.00 (ref) | 1.00 (ref) | 1.00 (ref) | 1.00 (ref) | 1.00 (ref) | 1.00 (ref) | 1.00 (ref) | 1.00 (ref) |
|  |  | High-level of household income | **0.79 (0.78 to 0.81)** | **0.85 (0.81 to 0.88)** | **0.87 (0.83 to 0.90)** | **0.90 (0.86 to 0.94)** | **0.91 (0.87 to 0.95)** | **0.86 (0.81 to 0.91)** | 0.91 (0.82 to 1.00) | **0.79 (0.71 to 0.88)** | **0.88 (0.80 to 0.96)** |
|  | Overweight | Low-level of household income | **1.26 (1.23 to 1.30)** | **1.29 (1.21 to 1.36)** | **1.30 (1.23 to 1.39)** | **1.25 (1.17 to 1.33)** | **1.19 (1.11 to 1.27)** | **1.30 (1.18 to 1.44)** | **1.18 (1.01 to 1.38)** | **1.38 (1.18 to 1.62)** | **1.52 (1.32 to 1.74)** |
|  |  | Mid-level of household income | 1.00 (ref) | 1.00 (ref) | 1.00 (ref) | 1.00 (ref) | 1.00 (ref) | 1.00 (ref) | 1.00 (ref) | 1.00 (ref) | 1.00 (ref) |
|  |  | High-level of household income | **0.81 (0.79 to 0.83)** | **0.85 (0.80 to 0.91)** | **0.86 (0.81 to 0.91)** | **0.92 (0.86 to 0.98)** | **0.93 (0.88 to 0.99)** | **0.86 (0.79 to 0.93)** | 0.88 (0.77 to 1.00) | 0.99 (0.87 to 1.13) | 0.93 (0.82 to 1.05) |
|  | Obese | Low-level of household income | **1.31 (1.28 to 1.34)** | **1.33 (1.26 to 1.40)** | **1.44 (1.36 to 1.52)** | **1.21 (1.14 to 1.28)** | **1.21 (1.14 to 1.28)** | **1.33 (1.22 to 1.44)** | **1.18 (1.04 to 1.34)** | **1.70 (1.50 to 1.93)** | **1.74 (1.54 to 1.97)** |
|  |  | Mid-level of household income | 1.00 (ref) | 1.00 (ref) | 1.00 (ref) | 1.00 (ref) | 1.00 (ref) | 1.00 (ref) | 1.00 (ref) | 1.00 (ref) | 1.00 (ref) |
|  |  | High-level of household income | **0.78 (0.76 to 0.80)** | **0.84 (0.79 to 0.89)** | **0.87 (0.82 to 0.92)** | **0.83 (0.78 to 0.88)** | **0.91 (0.86 to 0.96)** | **0.92 (0.86 to 0.98)** | **0.83 (0.74 to 0.93)** | **0.84 (0.75 to 0.95)** | **0.83 (0.75 to 0.92)** |
| Education | High school or lower education | Low-level of household income | **1.50 (1.47 to 1.53)** | **1.45 (1.40 to 1.51)** | **1.47 (1.42 to 1.53)** | **1.44 (1.38 to 1.50)** | **1.45 (1.39 to 1.52)** | **1.56 (1.47 to 1.65)** | **1.47 (1.33 to 1.62)** | **1.59 (1.44 to 1.75)** | **1.71 (1.57 to 1.87)** |
|  |  | Mid-level of household income | 1.00 (ref) | 1.00 (ref) | 1.00 (ref) | 1.00 (ref) | 1.00 (ref) | 1.00 (ref) | 1.00 (ref) | 1.00 (ref) | 1.00 (ref) |
|  |  | High-level of household income | **0.81 (0.78 to 0.85)** | **0.86 (0.79 to 0.94)** | **0.83 (0.76 to 0.90)** | **0.80 (0.72 to 0.90)** | **0.89 (0.80 to 0.99)** | 0.90 (0.81 to 1.01) | 0.88 (0.72 to 1.07) | 0.95 (0.77 to 1.19) | **0.64 (0.52 to 0.79)** |
|  | College or higher education | Low-level of household income | **1.21 (1.18 to 1.23)** | **1.22 (1.16 to 1.28)** | **1.22 (1.15 to 1.29)** | **1.16 (1.11 to 1.22)** | **1.14 (1.08 to 1.20)** | **1.32 (1.21 to 1.44)** | **1.19 (1.04 to 1.35)** | **1.53 (1.36 to 1.72)** | **1.68 (1.50 to 1.89)** |
|  |  | Mid-level of household income | 1.00 (ref) | 1.00 (ref) | 1.00 (ref) | 1.00 (ref) | 1.00 (ref) | 1.00 (ref) | 1.00 (ref) | 1.00 (ref) | 1.00 (ref) |
|  |  | High-level of household income | **0.78 (0.76 to 0.79)** | **0.83 (0.81 to 0.86)** | **0.87 (0.84 to 0.90)** | **0.88 (0.85 to 0.91)** | **0.90 (0.87 to 0.93)** | **0.85 (0.82 to 0.89)** | **0.83 (0.77 to 0.89)** | **0.83 (0.77 to 0.89)** | **0.88 (0.82 to 0.94)** |
| Smoking status | Smoker | Low-level of household income | **1.34 (1.30 to 1.38)** | **1.30 (1.24 to 1.38)** | **1.39 (1.30 to 1.48)** | **1.27 (1.19 to 1.35)** | **1.31 (1.22 to 1.40)** | **1.48 (1.33 to 1.64)** | **1.18 (0.99 to 1.40)** | **1.77 (1.50 to 2.10)** | **1.91 (1.65 to 2.20)** |
|  |  | Mid-level of household income | 1.00 (ref) | 1.00 (ref) | 1.00 (ref) | 1.00 (ref) | 1.00 (ref) | 1.00 (ref) | 1.00 (ref) | 1.00 (ref) | 1.00 (ref) |
|  |  | High-level of household income | **0.80 (0.78 to 0.82)** | **0.87 (0.82 to 0.92)** | **0.85 (0.80 to 0.90)** | **0.87 (0.81 to 0.93)** | **0.88 (0.83 to 0.94)** | **0.90 (0.83 to 0.97)** | **0.71 (0.62 to 0.82)** | 0.94 (0.81 to 1.08) | 0.93 (0.82 to 1.06) |
|  | Ex-smoker | Low-level of household income | **1.31 (1.27 to 1.36)** | **1.37 (1.28 to 1.46)** | **1.28 (1.19 to 1.38)** | **1.18 (1.09 to 1.27)** | **1.19 (1.10 to 1.28)** | **1.37 (1.24 to 1.52)** | **1.12 (0.92 to 1.35)** | **1.56 (1.29 to 1.87)** | **1.83 (1.57 to 2.13)** |
|  |  | Mid-level of household income | 1.00 (ref) | 1.00 (ref) | 1.00 (ref) | 1.00 (ref) | 1.00 (ref) | 1.00 (ref) | 1.00 (ref) | 1.00 (ref) | 1.00 (ref) |
|  |  | High-level of household income | **0.81 (0.78 to 0.84)** | **0.87 (0.80 to 0.94)** | **0.85 (0.79 to 0.92)** | **0.88 (0.81 to 0.96)** | 0.94 (0.86 to 1.01) | **0.90 (0.81 to 0.99)** | **0.83 (0.71 to 0.97)** | **0.77 (0.65 to 0.91)** | 1.01 (0.89 to 1.15) |
|  | Non-smoker | Low-level of household income | **1.33 (1.31 to 1.36)** | **1.34 (1.30 to 1.39)** | **1.42 (1.37 to 1.47)** | **1.30 (1.25 to 1.34)** | **1.24 (1.19 to 1.28)** | **1.43 (1.35 to 1.51)** | **1.29 (1.18 to 1.40)** | **1.42 (1.31 to 1.55)** | **1.54 (1.42 to 1.68)** |
|  |  | Mid-level of household income | 1.00 (ref) | 1.00 (ref) | 1.00 (ref) | 1.00 (ref) | 1.00 (ref) | 1.00 (ref) | 1.00 (ref) | 1.00 (ref) | 1.00 (ref) |
|  |  | High-level of household income | **0.79 (0.78 to 0.80)** | **0.83 (0.80 to 0.87)** | **0.88 (0.85 to 0.91)** | **0.89 (0.86 to 0.93)** | **0.93 (0.89 to 0.96)** | **0.86 (0.82 to 0.90)** | 0.94 (0.86 to 1.01) | **0.85 (0.78 to 0.92)** | **0.82 (0.76 to 0.89)** |
| Alcohol consumption | Non-drinker and 1day/month | Low-level of household income | **1.41 (1.38 to 1.43)** | **1.42 (1.38 to 1.47)** | **1.49 (1.44 to 1.54)** | **1.31 (1.27 to 1.36)** | **1.29 (1.24 to 1.34)** | **1.51 (1.44 to 1.59)** | **1.29 (1.18 to 1.40)** | **1.49 (1.37 to 1.61)** | **1.67 (1.54 to 1.80)** |
|  |  | Mid-level of household income | 1.00 (ref) | 1.00 (ref) | 1.00 (ref) | 1.00 (ref) | 1.00 (ref) | 1.00 (ref) | 1.00 (ref) | 1.00 (ref) | 1.00 (ref) |
|  |  | High-level of household income | **0.79 (0.77 to 0.80)** | **0.84 (0.80 to 0.88)** | **0.85 (0.82 to 0.89)** | **0.88 (0.84 to 0.92)** | **0.90 (0.86 to 0.95)** | **0.88 (0.83 to 0.93)** | **0.91 (0.83 to 0.99)** | **0.79 (0.73 to 0.86)** | **0.85 (0.78 to 0.92)** |
|  | 2–9 days/month | Low-level of household income | **1.25 (1.22 to 1.29)** | **1.23 (1.16 to 1.30)** | **1.23 (1.15 to 1.31)** | **1.21 (1.14 to 1.29)** | **1.19 (1.11 to 1.27)** | **1.34 (1.21 to 1.48)** | **1.22 (1.02 to 1.47)** | **1.60 (1.34 to 1.93)** | **1.72 (1.47 to 2.03)** |
|  |  | Mid-level of household income | 1.00 (ref) | 1.00 (ref) | 1.00 (ref) | 1.00 (ref) | 1.00 (ref) | 1.00 (ref) | 1.00 (ref) | 1.00 (ref) | 1.00 (ref) |
|  |  | High-level of household income | **0.79 (0.77 to 0.80)** | **0.84 (0.81 to 0.88)** | **0.88 (0.84 to 0.92)** | **0.89 (0.85 to 0.93)** | **0.92 (0.88 to 0.96)** | **0.85 (0.80 to 0.90)** | **0.80 (0.73 to 0.89)** | 0.92 (0.82 to 1.02) | **0.89 (0.81 to 0.98)** |
|  | Over 10 days/month | Low-level of household income | **1.12 (1.07 to 1.18)** | **1.11 (1.01 to 1.21)** | 1.01 (0.92 to 1.12) | 1.10 (1.00 to 1.22) | 1.11 (0.99 to 1.23) | 1.17 (1.00 to 1.37) | 0.89 (0.66 to 1.21) | **1.47 (1.11 to 1.95)** | **1.71 (1.28 to 2.28)** |
|  |  | Mid-level of household income | 1.00 (ref) | 1.00 (ref) | 1.00 (ref) | 1.00 (ref) | 1.00 (ref) | 1.00 (ref) | 1.00 (ref) | 1.00 (ref) | 1.00 (ref) |
|  |  | High-level of household income | **0.81 (0.78 to 0.85)** | **0.85 (0.76 to 0.95)** | **0.85 (0.77 to 0.95)** | 0.92 (0.82 to 1.03) | 0.94 (0.84 to 1.04) | **0.88 (0.78 to 0.99)** | 0.82 (0.65 to 1.04) | 0.95 (0.76 to 1.20) | 0.92 (0.74 to 1.16) |
| Health-level | High | Low-level of household income | 0.98 (0.94 to 1.02) | 1.04 (0.97 to 1.11) | 0.99 (0.91 to 1.08) | 0.96 (0.89 to 1.04) | 0.90 (0.82 to 0.98) | 0.96 (0.83 to 1.11) | 1.06 (0.88 to 1.28) | 1.09 (0.87 to 1.36) | **1.29 (1.04 to 1.60)** |
|  |  | Mid-level of household income | 1.00 (ref) | 1.00 (ref) | 1.00 (ref) | 1.00 (ref) | 1.00 (ref) | 1.00 (ref) | 1.00 (ref) | 1.00 (ref) | 1.00 (ref) |
|  |  | High-level of household income | **0.80 (0.78 to 0.82)** | **0.84 (0.80 to 0.89)** | **0.90 (0.86 to 0.95)** | **0.93 (0.88 to 0.99)** | **0.92 (0.87 to 0.97)** | **0.89 (0.82 to 0.96)** | **0.87 (0.78 to 0.96)** | 0.96 (0.86 to 1.07) | 0.95 (0.86 to 1.06) |
|  | Middle | Low-level of household income | **0.93 (0.90 to 0.95)** | 0.99 (0.94 to 1.03) | **0.94 (0.89 to 0.99)** | **0.90 (0.85 to 0.94)** | **0.85 (0.80 to 0.90)** | 0.99 (0.92 to 1.08) | **0.83 (0.73 to 0.94)** | 1.11 (0.98 to 1.26) | 1.11 (0.98 to 1.26) |
|  |  | Mid-level of household income | 1.00 (ref) | 1.00 (ref) | 1.00 (ref) | 1.00 (ref) | 1.00 (ref) | 1.00 (ref) | 1.00 (ref) | 1.00 (ref) | 1.00 (ref) |
|  |  | High-level of household income | **0.85 (0.84 to 0.87)** | **0.92 (0.88 to 0.96)** | **0.93 (0.90 to 0.97)** | **0.93 (0.89 to 0.97)** | 0.98 (0.94 to 1.02) | 0.96 (0.91 to 1.01) | 1.00 (0.91 to 1.10) | 0.94 (0.86 to 1.03) | 0.96 (0.89 to 1.05) |
|  | Low | Low-level of household income | **1.25 (1.23 to 1.28)** | **1.25 (1.21 to 1.30)** | **1.26 (1.21 to 1.31)** | **1.16 (1.12 to 1.21)** | **1.19 (1.14 to 1.24)** | **1.30 (1.23 to 1.38)** | 1.10 (0.99 to 1.23) | **1.26 (1.14 to 1.40)** | **1.48 (1.35 to 1.63)** |
|  |  | Mid-level of household income | 1.00 (ref) | 1.00 (ref) | 1.00 (ref) | 1.00 (ref) | 1.00 (ref) | 1.00 (ref) | 1.00 (ref) | 1.00 (ref) | 1.00 (ref) |
|  |  | High-level of household income | **0.92 (0.89 to 0.95)** | **0.89 (0.83 to 0.96)** | **0.93 (0.87 to 0.99)** | 0.95 (0.88 to 1.03) | 1.06 (0.99 to 1.15) | 1.03 (0.94 to 1.12) | 1.01 (0.85 to 1.21) | 0.88 (0.76 to 1.02) | 1.04 (0.90 to 1.21) |

**Abbreviations:** BMI, body mass index; CI, confidence interval; KCHS, Korea Community Health Survey; OR, odds ratios.

* According to the Asian–Pacific guidelines, the BMI is divided into four groups: underweight (<18.5 kg/m^2^), normal (18.5–22.9 kg/m^2^), overweight (23.0–24.9 kg/m^2^), and obese (≥25.0 kg/m^2^).

The numbers in bold indicate significant differences (p<0.05).
